# Completion of the cytosolic post-chorismate phenylalanine biosynthetic pathway in plants

**DOI:** 10.1038/s41467-018-07969-2

**Published:** 2019-01-03

**Authors:** Yichun Qian, Joseph H. Lynch, Longyun Guo, David Rhodes, John A. Morgan, Natalia Dudareva

**Affiliations:** 1 Department of Horticulture and Landscape Architecture, Purdue University, 625 Agriculture Mall Dr., West Lafayette, IN 47907-2010 USA; 2 Department of Biochemistry, Purdue University, 175 South University St., West Lafayette, IN 47907-2063 USA; 3 Davidson School of Chemical Engineering, Purdue University, 480 Stadium Mall Dr., West Lafayette, IN 47907-2100 USA; 4 Purdue Center for Plant Biology, Purdue University, West Lafayette, IN 47907 USA

## Abstract

In addition to being a vital component of proteins, phenylalanine is also a precursor of numerous aromatic primary and secondary metabolites with broad physiological functions. In plants phenylalanine is synthesized predominantly via the arogenate pathway in plastids. Here, we describe the structure, molecular players and subcellular localization of a microbial-like phenylpyruvate pathway for phenylalanine biosynthesis in plants. Using a reverse genetic approach and metabolic flux analysis, we provide evidence that the cytosolic chorismate mutase is responsible for directing carbon flux towards cytosolic phenylalanine production via the phenylpyruvate pathway. We also show that an alternative transcription start site of a known plastidial enzyme produces a functional cytosolic prephenate dehydratase that catalyzes the conversion of prephenate to phenylpyruvate, the intermediate step between chorismate mutase and phenylpyruvate aminotransferase. Thus, our results complete elucidation of phenylalanine biosynthesis via phenylpyruvate in plants, showing that this pathway splits from the known plastidial arogenate pathway at chorismate, instead of prephenate as previously thought, and the complete pathway is localized in the cytosol.

## Abstract (web summary)

Phenylalanine is mostly synthesized in plant plastids, but cytosolic transamination of phenylpyruvate also contributes. Here the authors show that a cytosolic chorismate mutase and a prephenate dehydratase encoded by an isoform of the plastidial ADT3 enzyme, produce phenylpyruvate in the cytosol.

## Introduction

Plants have a high demand for the aromatic amino acids L-phenylalanine, L-tyrosine, and L-tryptophan, as they serve as precursors for the formation of proteins and numerous aromatic primary and secondary metabolites^1^. Phenylalanine, for example, is a precursor of >8000 plant phenolic compounds, including the hormone salicylic acid^2^, quinones (ubiquinone)^3^, pigments (anthocyanins), aromatic volatiles^4,5^, phytoalexins, feeding deterrents (tannins), ultraviolet (UV) protectants (flavonoids), signal molecules (isoflavonoids)^6^, and structural components (lignin, suberin, and cell wall-associated phenolics)^6,7^. Broad physiological functions of these compounds and their importance for plant growth, development, reproduction, defense, and environmental responses explain the high carbon flux through the phenylalanine metabolic network^1^. In contrast to plants, animals lack the ability to synthesize phenylalanine and rely on a dietary supply. Moreover, phenylalanine-derived plant natural products possess biological activities and provide protection against a broad range of human diseases^8^. They are also used by humans as flavors, fragrances, biofuels, and insecticides^9^.

**Fig. 1:**
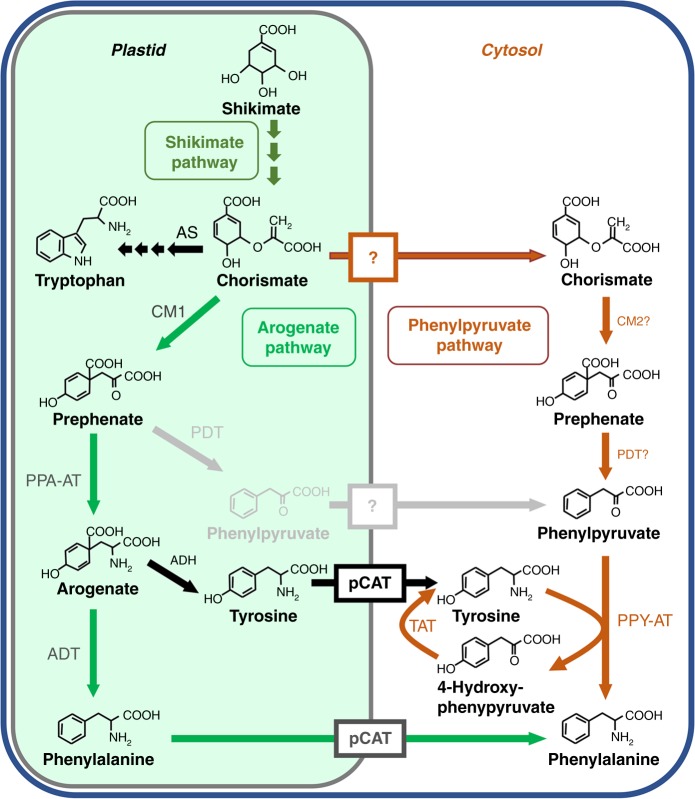
Proposed plant phenylalanine biosynthetic pathways. Characterized enzymes and transporters are shown in solid colors. Uncharacterized enzymes and transporters (boxes) are shown in gray or with question marks. ADH arogenate dehydrogenase, ADT arogenate dehydratase, AS anthranilate synthase, pCAT plastidial cationic amino acid transporter, CM chorismate mutase, PDT prephenate dehydratase, PPA-AT prephenate aminotransferase, PPY-AT phenylpyruvate aminotransferase, TAT tyrosine aminotransferase

In plants, biosynthesis of phenylalanine occurs via two alternative pathways, both requiring conversion of chorismate, the final product of the shikimate pathway, to prephenate by chorismate mutase. Genetic studies showed that out of the two pathways the major carbon flux is directed through the arogenate pathway^10–12^, which is initiated by transamination of prephenate to arogenate, followed by its decarboxylation/dehydration to phenylalanine (Fig. 1). In the alternative phenylpyruvate pathway, the order of reactions is reversed, and prephenate is first decarboxylated/dehydrated to phenylpyruvate, which is then transaminated to phenylalanine. Most microorganisms use the phenylpyruvate pathway to synthesize phenylalanine, but knowledge about this route in plants is still very fragmented^1,13^. The contribution of phenylpyruvate to phenylalanine biosynthesis has been demonstrated in Arabidopsis and petunia expressing a bacterial bifunctional chorismate mutase/prephenate dehydratase (CM/PDT) in plastids^14,15^. However, to date, no genes involved in this route have been identified in plants, except for a phenylpyruvate aminotransferase (PPY-AT) preferentially converting phenylpyruvate to phenylalanine^16^. While the enzymes involved in the arogenate pathway are localized in plastids, PPY-AT is instead localized in the cytosol^16^. This discovery raised the question of the origin of the phenylpyruvate used by the cytosolic PPY-AT in the microbial-like phenylpuryvate pathway in plants, and by extension, the subcellular localization of the entire pathway and its branching point from the known plastidial arogenate pathway.

It is possible that phenylpyruvate originates from the action of plastidial arogenate dehydratases (ADTs) with moonlighting PDT activity^10,17–19^ (Fig. 1). To date, six, nine, four, and three ADT genes have been identified in Arabidopsis, pine, rice and petunia, respectively^10,17–19^, encoding enzymes that are all localized in plastids^10,19,20^. Although some of these ADTs can use prephenate as substrate, the presence of prephenate aminotransferase (PPA-AT) converting prephenate to arogenate in the same subcellular compartment with catalytic efficiency that is at least 50-fold (Arabidopsis) to 900-fold (petunia) higher than that of respective ADTs with prephenate likely prevents formation of phenylpyruvate to support cytosolic phenylalanine biosynthesis (Fig. 1)^21^. Alternatively, formation of phenylpyruvate could take place in the cytosol, requiring a cytosolic pool of prephenate (Fig. 1).

Previously, it has been shown that a plastidial chorismate mutase (CM1) is predominantly responsible for phenylalanine production in petunia^22^. Nevertheless, for many years it has been known that plants also contain cytosolic chorismate mutase (CM2)^23–27^, but its physiological function remains unknown. The wide acceptance of plastids being the exclusive site of aromatic amino acid biosynthesis a priori excluded the consideration of CM2 involvement in phenylalanine biosynthesis but instead led to multiple proposals of alternative functions^22,23,28^. However, recent discovery of cytosolic PPY-AT^16^ and the widespread occurrence of cytosolic CM2s in plant species urge reconsideration of CM2 function and raise the prospect that plants contain a complete phenylpyruvate pathway for phenylalanine biosynthesis in the cytosol.

Here we used flowers of *Petunia hybrida* cv Mitchell, which have high carbon flux through the phenylalanine biosynthetic network and emit high levels of exclusively phenylalanine-derived volatiles^29,30^ to identify architecture and subcellular localization of the phenylpyruvate pathway for phenylalanine biosynthesis. By combining reverse genetic and metabolic flux analyses, we demonstrated that the cytosolic CM2 catalyzes the first step in the cytosolic phenylpyruvate pathway. We also show that the intermediate step between chorismate mutase and phenylpyruvate aminotransferase is catalyzed by prephenate dehydratase, the cytosolic localization of which is the result of transcription from an alternative transcription start site of a known gene encoding a plastidial enzyme. Together, these data complete elucidation of the microbial-like phenylpyruvate pathway in petunia and show that the whole pathway is localized in the cytosol.

## Results

### PhCM2 is required for cytosolic phenylalanine biosynthesis

Like all flowering plants^28^, *P. hybrida* contains both a plastidial CM (PhCM1) and a cytosolic CM (PhCM2)^22^. Biochemical characterization of both recombinant petunia enzymes revealed that, as it has been reported in Arabidopsis (*K*_m_ for chorismate 550 μM and 150 μM for AtCM1 and AtCM2, respectively^28^), PhCM2 has higher affinity towards chorismate (*K*_m_ for chorismate 174 μM and 8.6 μM for PhCM1 and PhCM2, respectively) (Table 1 and Supplementary Figure 1a and b). Moreover, both petunia enzymes are catalytically more efficient (*k*_cat_/*K*_m_) and differences between their catalytic efficiencies are more pronounced than those for Arabidopsis enzymes, likely reflecting the need to sustain the high flux towards phenylalanine and subsequently phenylalanine-derived volatiles in the floral tissue. Indeed, apparent catalytic efficiency of PhCM2 is 49.7-fold higher than that of PhCM1 (Table 1), while in Arabidopsis this difference is only 8.8-fold^28^.

**Table 1:** Kinetic parameters of recombinant petunia PhCM2

Enzyme	*K*_m_ (μM)	*V*_max_ (μmol/min/mg)	*k*_cat_/*K*_m_ (M/s)
PhCM1	174 ± 27	45.7 ± 1.5	144,000
PhCM2	8.6 ± 1.2	114 ± 4	7,136,000

Data are the mean ± SEM (*n* = 3)

A previous report of *PhCM1* RNA interference (RNAi) downregulation supports its in planta role in phenylalanine biosynthesis in plastids^22^; however, an in vivo function of PhCM2, or CM2 from any plant species, has not been established. To determine if *PhCM2* expression correlates with known phenylalanine biosynthetic genes, either the arogenate or phenylpyruvate pathways, *PhCM2* transcript levels were analyzed in petunia flowers by quantitative reverse transcription-PCR (qRT-PCR) with gene-specific primers. Like *PhPPY-AT*, *PhCM2* transcripts were found in all floral tissues and leaves (Supplementary Figure 2a) demonstrating that *PhCM2* expression does not exhibit spatial profile typical for genes involved in the arogenate pathway, *PhADT1* and *PhPPA-AT*^10,16,21^. However, similar to *PhADT1* and *PhPPA-AT*, *PhCM2* messenger RNA (mRNA) levels in corolla increased after flower opening, although to a lesser extent (Supplementary Figure 2b). In addition, *PhCM2* transcripts exhibited rhythmicity with the highest levels at 19:00 and 23:00 h (Supplementary Figure 2c), which corresponds to the peak of phenylalanine content^10^ and emission of phenylalanine-derived volatiles^29,30^ over a daily light/dark cycle.

**Fig. 2:**
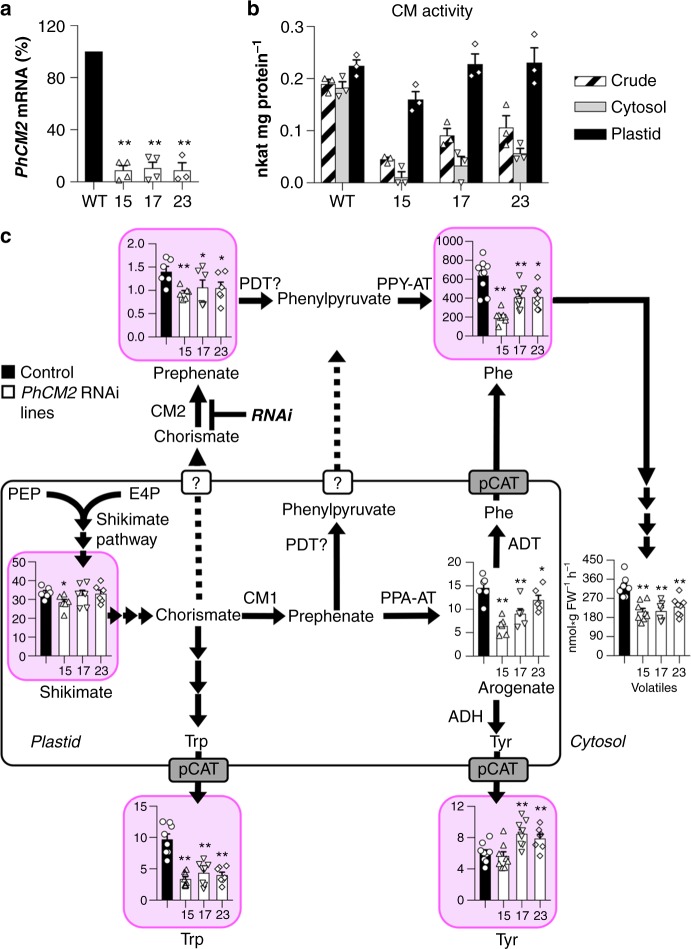
Metabolic effects of *PhCM2* RNAi downregulation in petunia flowers. **a** *PhCM2* mRNA levels in 2-day-old petunia petals at 20:00 h. Expression levels in transgenic lines (white bars) are shown as a percentage of *PhCM2* expression in control (black bar) petals set as 100% (*n* ≥ 3 biological replicates). **b** CM activities with 500 μM chorismate in crude extracts (striped bars), and cytosolic (gray bars) and plastidial (back bars) fractions prepared from corollas of 1- to 3-day-old wild type and *PhCM2* RNAi petunia flowers harvested at 10:00 h (*n* = 3 biological replicates). **c** Internal pools of shikimate, prephenate, arogenate, and aromatic amino acids as well as total volatile emission (*n* ≥ 6 biological replicates). Metabolite levels were measured in 2-day-old petunia petals at 20:00 h and are shown in nmol/gFW. Pink background indicates metabolites with potential dual subcellular localization, in the cytosol and plastids. Emitted volatiles were collected from 2-day-old wild-type and transgenic *PhCM2* RNAi petunia flowers from 18:00 h to 22:00 h. Black and white bars represent wild-type and transgenic lines, respectively. Data are means ± SE. *P < 0.05, **P < 0.01 as determined by paired two-tailed Student’s *t-*test. ADT arogenate dehydratase, ADH arogenate dehydrogenase, CM chorismate mutase, E4P erythrose 4-phosphate, pCAT plastidial cationic amino acid transporter, PDT prephenate dehydratase, PEP phospho*enol*pyruvate, PPA-AT prephenate aminotransferase, PPY-AT phenylpyruvate aminotransferase, FW fresh weight

To examine the in vivo function of PhCM2, its expression was down-regulated in petunia flowers using RNAi approach under the control of a petal-specific promoter^31^. Thirty independent transgenic lines were generated and three lines with the greatest decrease in *PhCM2* transcript levels (~90%) were selected for further analysis and metabolic profiling (Fig. 2a). RNAi suppression of *PhCM2* expression resulted in 66–81% reduction in CM activity detected in cytosolic fraction relative to wild type (Fig. 2b). It should be noted that the reduction in the cytosolic CM activity in transgenic lines is underestimated since cytosolic fraction contained some plastidial proteins, including PhCM1, based on PPA-AT activity used as plastidial marker (Supplementary Figure 3a). In plastids, which were free from cytosolic contamination based on alcohol dehydrogenase activity assays (Supplementary Figure 3b), CM activity remained unchanged and showed similar enrichment relative to crude extract (2.5-fold on average across *PhCM2*-RNAi lines) (Fig. 2b) as PPA-AT activity (2.4-fold on average across *PhCM2*-RNAi lines and wild type (Supplementary Figure 3a). Reduced *PhCM2* expression led to a decrease in endogenous phenylalanine levels in the petals by 33 to 64% compared to wild type (Fig. 2c), which was metabolically indistinguishable from empty vector control (Supplementary Figure 4). Transgenic flowers also emitted 30 to 37% less phenylalanine-derived volatiles relative to wild type (Fig. 2c), but the extent of decrease varied among the individual compounds (Supplementary Figure 5). In addition, tyrosine levels in the petals of *PhCM2* RNAi lines were increased by 31 to 41% at least in two transgenic lines relative to controls (Fig. 2c). Tyrosine is a known amino donor for PhPPY-AT^16^, an enzyme which is downstream of CM2 in the phenylpyruvate pathway (Fig. 1), and thus its accumulation is consistent with reduced flux towards cytosolic phenylalanine biosynthesis. In contrast to tyrosine, tryptophan levels were decreased by 52 to 65% in transgenics (Fig. 2c). At the same time, analysis of internal pools of shikimate and phenylalanine biosynthetic intermediates revealed that prephenate was reduced by 25 to 33% (Fig. 2c) and arogenate by 19 to 53% relative to controls, while shikimate remained unchanged except for transgenic line 15, which exhibited 15% reduction (Fig. 2c). The levels of phenylpyruvate remained below detection limit, while we were unable to detect chorismate in plant tissue.

### PhCM2 influences flux through both phenylalanine pathways

**Fig. 3:**
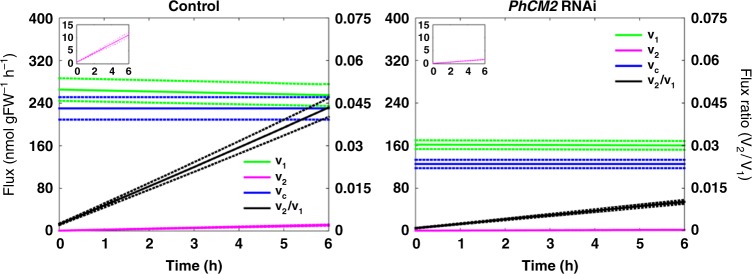
Metabolic modeling of phenylalanine biosynthetic pathways in control and *PhCM2* RNAi petunia flowers. Flux models representing the phenylalanine biosynthetic network in 2-day-old control and *PhCM2* RNAi petunia flowers. Metabolic modeling was performed using pool sizes and isotopic abundances of phenylalanine and tyrosine, and measurements of phenylalanine-derived volatile emission (consumption flux, *v*_c_, blue lines) from petunia petals supplied with 10 mM ^15^N-tyrosine for up to 6 h starting at 18:00 h (*n* = 3 biological replicates). *v*_1_ flux through the plastidial arogenate pathway (green lines), *v*_2_ flux through the cytosolic phenylpyruvate (pink lines, also is enlarged in inserts). The *v*_2_/*v*_1_ ratio is shown by black lines. Solid lines are estimated values and dotted lines are standard deviation for each flux value

We previously developed a strategy to determine the relative flux through the phenylpyruvate pathway based on the unique property of cytosolic PhPPY-AT to use tyrosine as its amino donor^16,32^. Since the plastidial PPA-AT is unable to use tyrosine^21^, the incorporation of ^15^N label into the amino group of phenylalanine from ^15^N-tyrosine allows assessment of flux through the phenylpyruvate pathway^16^. To determine the effect of *PhCM2*-RNAi downregulation on the carbon flux through the phenylalanine biosynthetic pathways, 2-day-old control and *PhCM2* RNAi line 17 flowers were fed with 10 mM ^15^N-tyrosine starting at 18:00 h. After 2, 4, and 6 h of feeding, phenylalanine and tyrosine pool sizes and their isotopic abundances were analyzed by liquid chromatography-mass spectrometry (LC-MS) and used in our recently developed metabolic flux model of the phenylalanine biosynthetic network^32^. As previously reported^16,32^, the labeling percentage of phenylalanine increased linearly over a 6 h period (Supplementary Figure 6). The model revealed that *v*_2_, the flux through the cytosolic phenylpyruvate pathway, was 76 and 85% lower (*p* < 0.0005, two-tailed Student’s *t*-test, *n* = 3, Bonferroni corrected) at *t*_0_ and *t*_6_, respectively, in *PhCM2* RNAi plants relative to control (Fig. 3 and Table 2), further supporting that PhCM2 contributes to phenylalanine biosynthesis via the cytosolic phenylpyruvate pathway. At the same time, the flux analysis showed that in *PhCM2-*RNAi line, *v*_1_, the flux through the plastidial arogenate pathway, was also decreased by 39 and 37% (*p* < 0.005, two-tailed Student’s *t*-test, *n* = 3, Bonferroni corrected) at *t*_0_, and *t*_6_, respectively, relative to control (Fig. 3 and Table 2). The decrease in plastidial aromatic amino acid biosynthesis is consistent with observed decrease in internal pools of arogenate and tryptophan (Fig. 2c).

**Table 2:** Changes in flux in flowers from control and *PhCM2-RNAi* petunia lines at t_0h_ and t_6h_

	Plastidial synthesis rate *v*_1_ (nmol/gFW/h)			Cytosolic synthesis rate *v*_2_ (nmol/gFW/h)		
	*t*_0h_	*t*_6h_	Relative change at *t*_6h_	*t*_0h_	*t*_6h_	Relative change at *t*_6h_
Control	266 ± 21.2	255 ± 20.5	−3.95%	0.627 ± 0.0560	11.1 ± 1.08	+1660%
*PhCM2* RNAi	162 ± 8.28	160 ± 8.22	−1.17%	0.147 ± 0.0168	1.64 ± 0.131	+993%
Relative change in *PhCM2* RNAi	−39.0%	−37.3%		−76.2%	−85.2%	

*v*_1_: Absolute flux through the plastidial arogenate pathway

*v*_2_: Absolute flux through the cytosolic phenylpyruvate pathway

(−): Indicates a decrease in flux at *t*_6h_ versus *t*_0h_ or in *PhCM2* RNAi versus control

(+): Indicates an increase in flux at *t*_6h_ versus *t*_0h_

The decreased flux through the arogenate pathway in *PhCM2* transgenics could be the result of a decreased consumption of cytosolic chorismate, accumulation of which could reduce export of chorismate from plastids, and redirecting it to the formation of aromatic amino acids in this organelle. If the resulting increase in plastidial phenylalanine and tyrosine pools then exceeds transport capability of PhpCAT, a plastidial aromatic amino acid transporter^32^, it would lead to their buildup, and inhibit upstream enzymes within the network. To test if such feedback regulation occurs and can be overcome by feeding of shikimate pathway intermediate, 2-day-old *PhCM2*-RNAi and control petunia petals were fed with 100 mM shikimate for 7 h starting at 15:00 h, and pool sizes of arogenate, prephenate and phenylpyruvate, as well as aromatic amino acids were analyzed. Feeding of shikimate increased internal pools of phenylalanine biosynthetic intermediates, arogenate, prephenate and phenylpyruvate, as well as tyrosine, in both genetic backgrounds such that there were no statistical differences between control and transgenics for any of those compounds after feeding except for arogenate, which still remained 30% lower in transgenics (Supplementary Figure 7). In contrast, phenylalanine and tryptophan were increased only in the *PhCM2* RNAi background, with phenylalanine, but not tryptophan, still remaining below wild-type levels. These results indicate that increased availability of shikimate recovers the plastidial aromatic amino acid flux in the *PhCM2* RNAi line to wild-type levels, while cytosolic flux remains depressed. A decrease in the cytosolic flux persists despite a dramatic accumulation in phenylpyruvate. This may indicate that phenylpyruvate is being formed within the plastid by PDT activity of ADTs and is inaccessible to the cytosolic PhPPY-AT due to limited transport out of plastids.

To determine whether a reduced flux through the arogenate pathways is the result of transcriptional regulation of: (i) 3-deoxy-d-*arabino*-heptulosonate 7-phosphate synthase (DAHPS), which catalyzes the first committed step in the shikimate pathway^33,34^; (ii) other genes encoding key enzymes of the shikimate pathway (e.g., 5-enolpyruvylshikimate 3-phosphate synthase (EPSPS) and CM1), or (iii) petunia shikimate pathway transcriptional regulator ODORANT1 (ODO1)^35^, their transcript levels were analyzed in petals of transgenic and control plants using qRT-PCR. This analysis revealed that expression of these genes remained unchanged in *PhCM2*-RNAi plants relative to control (Supplementary Figure 8a). Likewise, activities of PPA-AT and ADT in plastids were unaffected by *PhCM2* downregulation (Supplementary Figures 3a and 8b).

To assess whether feedback regulation within the plastidial aromatic amino acid network can be overcome by an increased capacity of a plastidial Phe transporter, *PhpCAT* was transiently overexpressed in *PhCM2*-RNAi flowers and compared to that in wild-type control. *PhpCAT* overexpression led to a significant increase in phenylalanine levels and emission of phenylalanine-derived volatiles in both backgrounds (Supplementary Figure 9), demonstrating that the increased consumption flux of phenylalanine corresponds to an increase in its plastidial biosynthesis, supporting the feedback regulation hypothesis.

### CM2 contributes to phenylalanine biosynthesis in Arabidopsis

**Fig. 4:**
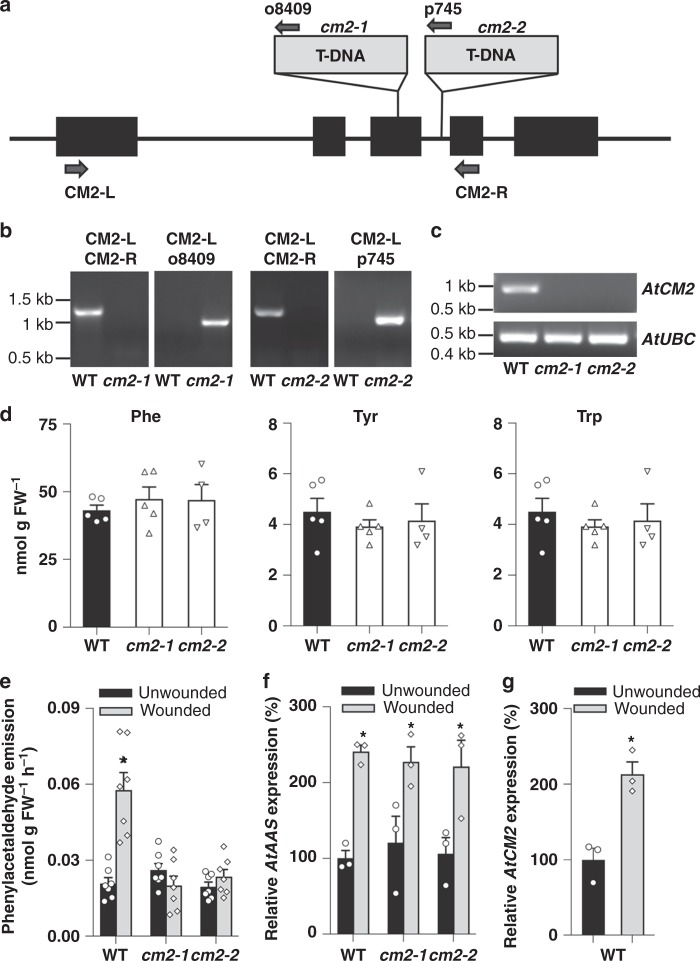
Characterization of the Arabidopsis *cm2* mutants. **a** Structure of the *AtCM2* gene showing the T-DNA insertion sites in *cm2-1* and *cm2-2* mutant lines. Black boxes represent exons. Arrows indicate the positions of primers used for analysis. **b** Genotyping of the Arabidopsis *cm2* mutants by PCR analysis using primers specific to genomic regions flanking each T-DNA as well as T-DNA-specific primers. **c** RT-PCR analysis of *AtCM2* transcripts in wild-type (ecotype Col-0) and the *cm2* mutants. Transcript levels of *AtUBC* encoding ubiquitin-conjugating enzyme were used as endogenous control. **d** Aromatic amino acid levels in leaves of Arabidopsis wild-type (ecotype Col-0; black bars) and *cm2* mutants (white bars). Data are means ± SE (n ≥ 3 biological replicates). **e** Phenylacetaldehyde emission from leaves of Arabidopsis wild-type and *cm2* mutants upon mechanical wounding. Volatiles were collected from intact (black bars) and wounded (gray bars) leaves for 24 h and analyzed by GC-MS. Data are means ± SE (*n* ≥ 7 biological replicates). *P *<* 0.05 as determined by paired two-tailed Student’s *t*-test. **f** *AtAAS* transcript levels in Arabidopsis leaves of wild-type and *cm2* mutants upon mechanical wounding, as determined by qRT-PCR. **g** *AtCM2* transcript levels in wild-type Arabidopsis leaves upon mechanical wounding, as determined by qRT-PCR. In (**f**, **g**) data are presented as relative to untreated wild-type control plants set as 100%, and bar colors are same as in (**e**). Data are means ± SE (*n* = 3 biological replicates). *P *<* 0.05 as determined by paired two-tailed Student’s *t*-test

To determine whether CM2 is involved in phenylalanine biosynthesis via the phenylpyruvate pathway in other plant species, we searched TAIR (The Arabidopsis Information Resource) database for Arabidopsis *AtCM2* T-DNA insertion mutants. Two T-DNA insertion lines were identified: N414383 (*cm2-1*) line, carrying a T-DNA insertion within the 3rd exon of the *AtCM2* gene (At5g10870), and CS849985 (*cm2-2*) line with a T-DNA insertion in the 3rd intron (Fig. 4a). The genotypes of obtained homozygous *cm2* plants were confirmed by genomic PCR analysis with gene-specific primers flanking the insertion sites, which failed to amplify the respective gene regions (Fig. 4b). No *AtCM2* transcripts were detected in *cm2* mutants by RT-PCR (Fig. 4c). Analysis of aromatic amino acid internal pools in leaves revealed no differences between *cm2* mutants and wild-type plants (Fig. 4d). We have previously shown that mechanical wounding of Arabidopsis leaves increases emission of phenylacetaldehyde, which is formed directly from phenylalanine by aromatic aldehyde synthase (AtAAS), a bifunctional enzyme that catalyzes phenylalanine decarboxylation/deamination^36^. While mechanical wounding increased phenylacetaldehyde emission in wild-type leaves, there were no changes in phenylacetaldehyde emission in wounded leaves of either *cm2-1* or *cm2-2* mutants, levels of which were similar to that in unwounded wild-type leaves (Fig. 4e). Analysis of *AtAAS* expression by qRT-PCR revealed that its transcript levels were increased to the same degree in *cm2-*mutant as in wild-type leaves upon wounding (Fig. 4f). Furthermore, wounding of wild-type leaves increased expression of *AtCM2* by twofold (Fig. 4g). Taken together, these results indicate that AtCM2 contributes to phenylalanine biosynthesis and subsequently to phenylpropanoid metabolism in Arabidopsis, especially when demand for phenylpropanoids is increased as a part of plant defense.

### Alternative transcription produces cytosolic PhADT3 isoform

In the cytosol, PhCM2 produces prephenate and PhPPY-AT converts phenylpyruvate to phenylalanine, and thus we hypothesized that the conversion of prephenate to phenylpyruvate by PDT takes place in the same subcellular compartment (Fig. 1). To date, PDT activity has been detected only in etiolated Arabidopsis seedlings^37^. Since PDT activity was below detection limit when analyzed in cytosolic fraction obtained from petal tissue, petunia petals were subjected to non-aqueous fractionation and PDT activity as well as activities of compartment-specific marker enzymes (phospho*enol*pyruvate carboxylase (PEPC) and NADPH-dependent glyceraldehyde-3-phosphate dehydrogenase (GAPDH) for cytosol and plastid, respectively) were analyzed within the gradient fractions to assess subcellular enrichment (Supplementary Figure 10), which was determined using best fit algorithm^38^. In parallel, activities of several enzymes with established subcellular localizations were used to validate this approach. Indeed, phenylalanine ammonia lyase (PAL)^39^, PPA-AT^12^, and PPY-AT^16^ were correctly found to be localized in the cytosol, plastids, and cytosol, respectively (Supplementary Figures 10 and 11). Moreover, CM activity in the presence of 20 µM and 500 µM chorismate was predominantly distributed in the cytosol and plastids, respectively, consistent with high and lower affinity of petunia cytosolic and plastidial CM isoforms for this substrate (Table 1). Plastidial, but not cytosolic, CMs have previously been shown to be activated by tryptophan^28^. Assays performed in the presence of 1 mM tryptophan resulted in almost exclusive plastidial localization of CM activity (Supplementary Figures 10 and 11). Analysis of PDT activity revealed a split at nearly a 1:1 ratio between cytosol and plastids (Supplementary Figures 10 and 11), the latter is likely due to PDT activities of plastidial PhADT2 and PhADT3^10^.

To date, no dedicated PDTs have been identified in plants, although certain ADTs from Arabidopsis, rice, pine, and petunia (PhADT2 and PhADT3) have been shown to utilize prephenate as substrate^10,17–19^. Recent studies in pine identified, outside of ADT/PDT catalytic domain, a 22-amino acid PDT activity conferring (PAC) domain in which a single residue Ala314 is thought to trigger PDT activity based on yeast complementation assays^19^. Out of two petunia ADTs displaying PDT activities in vitro^10^, only PhADT2 retained this residue. Therefore, to test for bona fide PDT activities of petunia ADTs, complementation of the *Saccharomyces cerevisiae pha2* mutant, which lacks PDT activity and is unable to grow without phenylalanine supplementation, was performed. Both PhADT2 and PhADT3 were able to restore growth of *pha2* mutant, which reached stationary phase after 2 and 4 days, respectively (Supplementary Figure 12), while yeast strain expressing PhADT1 displayed significant delay in growth. Consistent with our previously reported catalytic efficiencies of recombinant petunia ADTs^10^, these results confirm that PhADT2 and PhADT3 possess PDT activity in vivo, and additional factors beyond Ala314 are involved in determining substrate specificity.

**Fig. 5:**
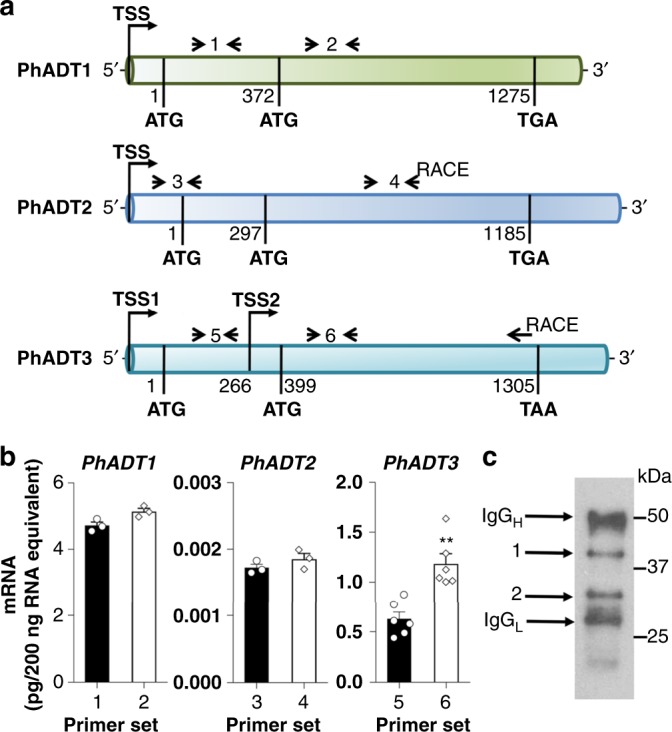
Alternative *PhADT3* transcript yields a cytosolic isoform in petunia flowers. **a** Schematic presentation of *PhADT1*, *PhADT2,* and *PhADT3* mRNA and positions of gene-specific primer sets shown in Supplementary Table 1. The translation initiation codons as well as stop codons are indicated in bold. Nucleotide positions are referred to the first translation initiation codon. TSS transcription start site, RACE depicts the position of primers used for 5′ -RLM-RACE. **b** Alternative *PhADT3* transcripts in petunia flowers. The absolute transcript levels of *PhADT1*, *PhADT2*, and *PhADT3* were determined at 5′ end (black bars) and 3′ end (white bars) of mRNAs by qRT-PCR and are shown as pg/200 ng total RNA. mRNA was obtained from petunia flowers harvested at 20:00 h on day 2 post anthesis. Data are means ± SE (*n* = 3 biological replicates for *PhADT1* and *PhADT2*; *n* = 6 biological replicates for *PhADT3*). **P < 0.01 as determined by paired two-tailed Student’s *t*-test. **c** Representative immunoblot (out of seven) using anti-PhADT3 antibodies against PhADT3 immune complex prepared from 1 mg protein from crude extract of 2-day-old petunia petals harvested at 10:00 h. Then, 4 µl of isolated PhADT3 immune complex was loaded on a gel. Regions corresponding to bands 1 and 2 of the SDS-PAGE gel were excised and used for proteomics analysis. IgG_H_ and IgG_L_ correspond to the heavy and light chains of IgG, respectively

All characterized ADTs, however, contain N-terminal transit peptides targeting the proteins to plastids^10,19,20^. Even so, dual subcellular localization of proteins often could be achieved by using two alternative in-frame translation initiation codons, where the isoform produced from the first methionine possesses a plastid-targeting peptide while a truncated isoform generated from the second methionine could be targeted to the cytosol^40–43^. Examination of protein sequences revealed that PhADT2 and PhADT3 each contain a second in-frame ATG codon located 99 and 133 amino acids downstream from their respective first translation initiation codon (Fig. 5a).

Previously, assemblies of overlapping expressed sequence tag (EST) sequences with homology to Arabidopsis ADTs resulted in identification of *PhADT2* and *PhADT3* transcripts, which contain full-length open reading frames and respective 5′-untranslated regions (UTRs) of 176 and 121 bp in size^10^. To test for a second transcription start site (TSS) for these transcripts, the 5′-RNA ligase-mediated rapid amplification of the cDNA ends (5′-RLM-RACE) was performed using RNA extracted from 2-day-old petals at 20:00 h and gene-specific primers. The use of the GeneRacer^TM^ Kit (Invitrogen) enables the amplification of only full-length transcripts while eliminating truncated (5′ degraded) messages from the amplification process. 5′-RLM-RACE with *PhADT2*-specific primer designed downstream of the second in-frame ATG codon produced a PCR product of ~0.9 kb in size, the sequence of which corresponded to 5′-UTR already recovered from EST sequences. There were no PCR products between 450 and 747 bp in length corresponding to the putative TSS located between first and second ATG codons (Fig. 5a). In contrast, *PhADT3*-specific primer designed downstream of the second in-frame ATG codon produced a PCR fragment of about 1 kb in size. Sequencing of this fragment revealed the existence of a second TSS (TSS2) located 133 bp upstream of the second ATG codon (Fig. 5a). A potential TATA box was identified using Plant-CARE program^44^ 29 bp upstream from this TSS2. The shorter *PhADT3* transcript, designated as *PhADT3S*, encodes PhADT3S isoform containing intact ADT/PDT catalytic and ACT regulatory domains, but lacking a plastid-targeting peptide (Supplementary Figure 13a).

To independently test for the presence of shorter *PhADT3* transcripts, qRT-PCR was performed with two pairs of gene-specific primers targeted to different regions of the complementary DNA (cDNA) generated from RNA extracted from petals at 20:00 h on day 2 post anthesis, the time with the highest expression of *PhADTs*^10^. One set of gene-specific primers was designed upstream of the TSS2 (set 5) to detect only the long transcripts, designated *PhADT3L*, encoding long PhADT3 isoform (PhADT3L), and the other (set 6) was situated downstream of the second ATG codon to detect the entire *PhADT3* transcript population (*PhADT3L* plus *PhADT3S*) (Fig. 5a). Both pairs of primers were found to have similar efficiencies (>99%) based on amplification from a dilution series of purified full-length *PhADT3* template. The absolute amount of *PhADT3* transcripts obtained with set 6 primers (1.14 pg/200 ng RNA equivalent) was almost 2-fold higher than that with set 5 primers (0.62 pg/200 ng RNA equivalent) (Fig. 5b), suggesting that both types of transcripts, *PhADT3L* and *PhADT3S*, are produced in the cell at nearly similar levels. In contrast, similar experiment targeting *PhADT2* with sets 3 and 4 of *PhADT2*-specific primers revealed no differences (Fig. 5a and b), consistent with 5′-RLM-RACE data showing the absence of alternative TSS. In line with these experiments, *PhADT1* was included despite not possessing PDT activity. Similar to *PhADT2*, one type of transcript was detected for *PhADT1* with *PhADT1*- specific primer sets 1and 2 (Fig. 5a and b). Previously, it has been shown that *PhADT3* is expressed in petunia flowers and exhibits rhythmic expression corresponding to production of phenylalanine and emission of phenylalanine-derived volatiles^10^. Interestingly, both *PhADT3L* and *PhADT3S* display similar rhythmicity in their expression patterns over a day/night cycle (Supplementary Figure 14).

To experimentally determine whether the *PhADT3S* transcript encodes a cytosolically localized protein, its coding region was fused to either N or C terminus of the green fluorescent protein (GFP) reporter coding sequence. The resulting constructs were transiently expressed in *Nicotiana benthamiana* leaves along with GFP as a positive cytosolic control^45^. When samples were analyzed by confocal laser scanning microscopy, GFP fluorescence for all fusion proteins was found in the cytosol (Supplementary Figure 15), thus confirming the predicted cytosolic localization of PhADT3S.

To verify if the cytosolic PhADT3S isoform is catalytically active, the recombinant protein starting from a second ATG codon as well as PhADT3L (Supplementary Figure 13a) were produced in *Escherichia coli*, purified and found to retain dehydratase activities with both prephenate and arogenate substrates. Further analysis of PDT activity revealed that both isoforms have similar affinity for prephenate (*K*_m_ for prephenate 358 ± 32 μM and 433 ± 73 μM for PhADT3L and PhADT3S, respectively), while the apparent catalytic efficiency of PhADT3S is slightly (1.6-fold) lower than that of PhADT3L (Table 3 and Supplementary Figure 1c and d).

**Table 3:** Kinetic parameters of recombinant petunia PhADT3 isoforms with prephenate as substrate

Enzyme	*K*_m_ (μM)	*V*_max_ (μmol/min/mg)	*k*_cat_/*K*_m_ (M/s)
PhADT3L	358 ± 32	0.125 ± 0.060	252
PhADT3S	433 ± 73	0.113 ± 0.006	186

Data are the mean ± SEM (*n* = 3)

In silico sequence analysis predicted that the sizes of mature PhADT3L protein (i.e., without transit peptide that is cleaved in vivo) and PhADT3S are 42 kD and 33 kD, respectively (Supplementary Figure 13a). To experimentally determine if in planta alternative TSSs in *PhADT3* produce PhADT3 isoforms with different sizes and subsequently different subcellular localization, we performed immunoprecipitation of PhADT3 proteins followed by immunoblot analysis with anti-PhADT3 antibodies. Antibodies generated against synthetic PhADT3 peptide, localized after the second methionine, recognized both recombinant long mature and short PhADT3 proteins and did not cross hybridize with the PhADT1 (Supplementary Figure 13b), the ADT protein with the highest expression and homology to PhADT3 (84% amino acid identity) in petunia petals^10^. Western blot analysis of the PhADT3 proteins immunoprecipitated from crude protein extracts prepared from 2-day-old petunia flowers revealed two bands of ~42 kD and ~33 kD in size (Fig. 5c), which were nearly equally abundant, consistent with their transcript levels (Fig. 5b) and subcellular distribution of detected PDT activity (Supplementary Figure 11). Proteomic analysis revealed the presence of PhADT3-specific peptides in both bands, indicating the existence of two PhADT3 isoforms in petunia flowers.

**Fig. 6:**
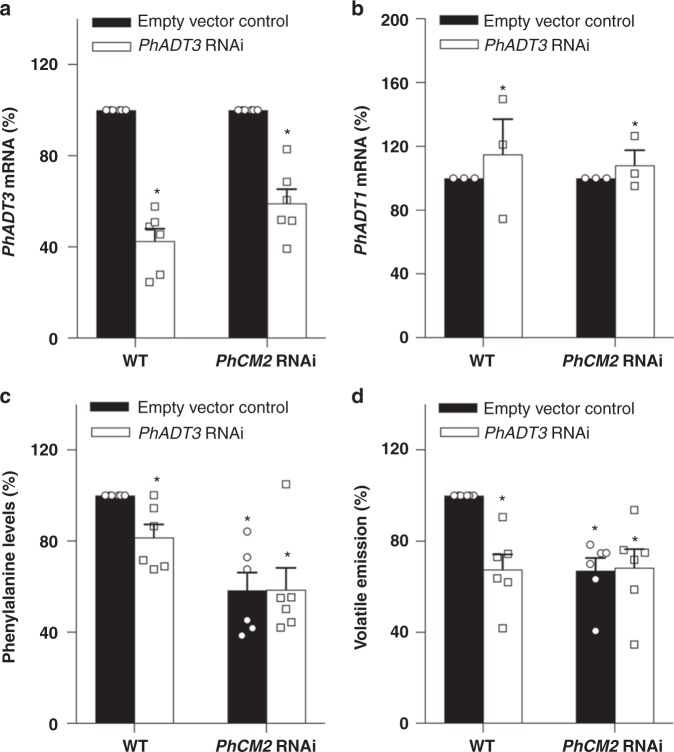
Effect of *PhADT3* RNAi downregulation in wild-type and *PhCM2* RNAi petunia flowers. **a**, **b** *PhADT3* mRNA (**a**) and *PhADT1* mRNA (**b**) levels in wild-type and *PhCM2* RNAi line 15 petunia flowers. For each genetic background, black and white bars represent flowers infiltrated with empty vector or the *PhADT3* RNAi construct, respectively. Data are presented as a percentage relative to the corresponding empty vector reference. **c**, **d** Levels of phenylalanine (**c**) and phenylalanine-derived volatiles (**d**) in petunia flowers of wild-type and *PhCM2* RNAi line 15 infiltrated with agrobacterium carrying the empty vector (black bars) or the *PhADT3* RNAi construct (white bars). For (**c**, **d**) data are presented as a percentage relative to the WT empty vector reference. Data are means ± SE (*n* = 6 biological replicates). *P *<* 0.01 as determined by paired two-tailed Student’s *t*-test

To further determine whether PhADT3 is involved in cytosolic phenylalanine biosynthesis in planta, its expression was transiently down-regulated in flowers of wild-type and *PhCM2* RNAi line 15. Reduction by 41–57% in *PhADT3* expression without an effect on *PhADT1* mRNA levels led to a decrease in phenylalanine levels and phenylalanine-derived volatiles by 19 and 35%, respectively, in wild-type flowers but did not impact their levels in *PhCM2* RNAi background (Fig. 6). Taken together, these results suggest that PhADT3 does indeed act in series with PhCM2 within the same phenylalanine biosynthetic pathway.

## Discussion

In plants, up to 30% of photosynthetically fixed carbon is directed to phenylalanine biosynthesis^46^, which can occur via two alternative routes (Fig. 1). Genetic evidence suggests that the majority of this carbon flux proceeds via the plastidial arogenate pathway^10–12^, discovery of which was only recently completed^21,47^. However, until recently, it remained unknown whether the alternative microbial-like phenylpyruvate route also contributes to phenylalanine biosynthesis. Evidence for the presence of a functional alternative route to phenylalanine production in plants first came from experiments in petunia flowers^16^. While downregulation of either of two consecutive steps in the arogenate pathway, catalyzed by PhADT1 and PhPPA-AT, resulted in reduced phenylalanine chemotype, their simultaneous downregulation restored phenylalanine to wild-type levels^16^. The subsequent isolation of a gene encoding a cytosolic PhPPY-AT, which transaminates phenylpyruvate to phenylalanine^16^, further supported the existence of alternative route, although it still remained unclear where the substrate for PhPPY-AT is synthesized.

Most microorganisms utilize only the phenylpyruvate pathway^48–50^ with a few exceptions^51^ and contain a bifunctional CM/PDT, which produces phenylpyruvate. In contrast, flowering plants contain multiple monofunctional CMs, with at least one isoform always being localized in the cytosol in species in which CMs have been analyzed^1,52^. The first gene encoding a cytosolic CM isoform in Arabidopsis (*AtCM2)* was isolated nearly two decades ago^23^, but the physiological role of the corresponding enzyme still remained unknown. The results presented here show that downregulation of *PhCM2* expression in petunia flowers leads to reduction of flux via the phenylpyruvate pathway (Fig. 3 and Table 2) with a concomitant decrease in the levels of phenylalanine and phenylalanine-derived volatile compounds (Fig. 2c), providing direct in vivo evidence for the involvement of PhCM2 in phenylalanine biosynthesis via the microbial-like phenylpyruvate route.

The contribution of a cytosolic CM to phenylalanine biosynthesis is not unique to petunia flowers. Under normal growth conditions, in leaves of wild-type and *cm2* Arabidopsis mutants there are no differences in phenylalanine internal pools (Fig. 4d) or in emission of phenylacetadehyde (Fig. 4e). However, in response to mechanical wounding, only in leaves of wild type, but not *cm2* mutants, emission of phenylacetadehyde is increased by 2.3-fold relative to unwounded controls (Fig. 4e). As phenylacetadehyde is derived directly from phenylalanine by AtAAS and *AtAAS* expression is similar in wild-type and *cm2*-mutant wounded leaves (Fig. 4f), these results suggest that *cm2* mutants are unable to produce substrate for AtAAS and that the excess of phenylalanine required for the phenylacetadehyde production upon wounding is produced by the phenylpyruvate route.

The cytosolic production of prephenate by PhCM2 and conversion of phenylpyruvate to phenylalanine by PhPPY-AT in the same subcellular compartment implies the existence of a functional cytosolic PDT converting prephenate to phenylpyruvate (Fig. 1). Alternatively, phenylpyruvate could be synthesized in plastids and transported to the cytosol. In general, phenylpyruvate is undetectable in most plant tissues^10,37^, but its level drastically increases upon shikimate feeding in petunia petals (Supplementary Figure 7 and^10^). Despite the fact that cytosolic phenylpyruvate production is blocked at CM2, feeding of *PhCM2*-RNAi flowers with exogenous shikimate increased the phenylpyruvate pool to the same level as in wild-type flowers, yet it did not lead to recovery of phenylalanine levels (Supplementary Figure 7). The reason for its inability to be converted to phenylalanine is currently unknown but could be due to a plastidial localization of this phenylpyruvate pool, where it is inaccessible to the cytosolic PhPPY-AT. We do not exclude that under shikimate feeding, as well as under overexpression of a bacterial CM/PDT in Arabidopsis and petunia plastids^14,15^, phenylpyruvate could be converted to phenylalanine in plastids by unknown aminotransferase(s) with low affinity towards phenylpyruvate or by moonlight activities of some aminotransferase(s). Nevertheless, this activity was unable to bypass the blockage in the cytosolic pathway in *PhCM2*-RNAi flowers and recover phenylalanine levels (Supplementary Figure 7).

To date, PDT activity has rarely been reported in plant tissues^37^. While monofunctional PDTs exist in yeast, fungi, and bacteria^53,54^, no such dedicated enzymes have been identified in plants. Moreover, only some of characterized plant ADTs utilize prephenate as substrate in addition to arogenate^10,17,19,53^. All characterized and putative plant ADTs/PDTs contain an N-terminal transit peptide targeting the proteins to plastids^10,17,19,20^. However, using non-aqueous fractionation, we show that PDT activity exists in the cytosol (Supplementary Figure 11). One of the mechanisms to direct the same protein to two different subcellular locations is the use of multiple TSSs to produce two in-frame proteins differing only in their N termini^40–42^. Recent large-scale full-length cDNA and genome sequence analyses in yeast^55^, humans^56^, and plants^57,58^ predicted that significant fraction of genes likely contains alternative TSS(s). 5′-RLM-RACE, qRT-PCR with two sets of primers upstream and downstream of the alternative TSS (Fig. 5a, b), and proteomics analysis of immunoprecipitated PhADT3 separated on and extracted from the sodium dodecyl sulfate-polyacrylamide gel electrophoresis (SDS-PAGE; Fig. 5c) revealed that PhADT3 contains an alternative TSS located 134 bp upstream of its second ATG codon (Fig. 5a), which encodes a functional PhADT3S isoform with PDT activity (Table 3) lacking the N-terminal transit peptide and thus localized in the cytosol (Supplementary Figure 15). Moreover, RNAi downregulation of *PhADT3* had no effect on levels of phenylalanine and phenylalanine-derived volatiles in *PhCM2* RNAi petunia flowers while reducing their levels in wild type (Fig. 6), further supporting the participation of PhADT3 in the cytosolic phenylpyruvate pathway.

Arabidopsis encodes six ADTs, with AtADT1, AtADT2, and AtADT6 being capable of utilizing prephenate as substrate in vitro^17^ and AtADT1 and AtADT2 furthermore being able to complement the *Saccharomyces cerevisiae* PDT-deficient *pha2* knockout mutant^53^. Similar to PhADT3, these three Arabidopsis proteins have in-frame ATG start codon located 86, 68, and 76 amino acids downstream from the first translation initiation codon in AtADT1, AtADT2, and AtADT6, respectively, but upstream of PDT/ADT catalytic domain. In silico analysis of corresponding genes using the Plant Promoter Databases^59^; www.softberry.com) predicted that *AtADT6*, but not *AtADT1* or *AtADT2*, contains two alternative TSS(s), suggesting that, similar to petunia, Arabidopsis could contain a cytosolic enzyme with PDT activity. Indeed, qRT-PCR with two pairs of *AtADT6* gene-specific primers revealed that region of cDNA downstream of predicted alternative TSS is 6.6-fold more abundant than upstream region, further suggesting that two types of *AtADT6* transcripts exist in Arabidopsis (Supplementary Figure 16a and b). Moreover, similar to *AtCM2*, expression of both *AtADT6* transcripts was upregulated by wounding, with a more profound effect on *AtADT6S* (Supplementary Figure 16c and d). Recently, the existence of different subcellular localizations, cytosolic and plastidial, was shown for AtADT3 depending on developmental stage and light conditions^60^. However, neither the mechanism of such dual localization nor the precise biochemical function in the cytosol of AtADT3, which does not possess PDT activity, were established. Interestingly, in silico analysis of all nine ADT genes previously identified in gymnosperm *Pinus pinaster*, four of which encode proteins with PDT activity in vitro^19^, using the Plant Promoter Databases, revealed that none of them contain alternative TSS capable of producing a cytosolically localized protein. A search of the SustainPine database^61^ using Arabidopsis CMs as queries identified two CMs (unigenes 37772 and 37813), both of which contain N-terminal plastid transit peptides based on TargetP. The absence of both cytosolically localized CM and ADT transcripts is consistent with a lack of the cytosolic phenylpyruvate pathway in gymnosperms, which requires further experimental validation. Likewise, the apparent lack of cytosolic CM isoforms in mosses and ferns^28^, suggests non-seed plants also lack the cytosolic phenylpyruvate pathway, consistent with a gain of the pathway in the angiosperm lineage, rather than the loss of an ancestral pathway in gymnosperms.

The discovery of CM2 function and a cytosolically localized enzyme with PDT activity in petunia completes the elucidation of the microbial-like phenylpyruvate route for phenylalanine biosynthesis. It also shows that the phenylpyruvate pathway branches from the known plastidial arogenate pathway at chorismate, and not at prephenate as previously thought, and that the entire pathway is localized in the cytosol. This discovery reinforces the long-standing suggestion that some plant species may possess a cytosolic pre-chorismate shikimate pathway, as extra-plastidic DAHPS and EPSPS isoforms as well as a gene encoding cytosolically localized 3-dehydroquinate dehydratase/shikimate dehydrogenase (DHD-SDH) have been reported (reviewed in ref. ^52^).

Given that the major carbon flux to phenylalanine is flowing via the arogenate pathway, an open question is why plants contain a functional phenylpyruvate route. The plastidial arogenate pathway in plants is subjected to complex feedback regulation at the entry and branch points catalyzed by CM1 and ADT, respectively^1,13,62^. In contrast, the phenylpyruvate route is subject to less stringent feedback regulation, because CM2, catalyzing the entry step in the phenylpyruvate pathway, is insensitive to allosteric regulation by aromatic amino acids^28^.

One remaining question about the cytosolic pathway is how significant it is in planta. Data available in petunia flowers show that the phenylpyruvate pathway can compensate for the phenylalanine production when the major route is impaired^16^ and for the shortage of phenylalanine when phenylalanine export out of plastids is impeded^32^. The blockage of the cytosolic phenylpyruvate pathway at CM2 causes a reduction in flux through the plastidial arogenate route (Fig. 3 and Table 2) via yet unknown mechanism. This effect points to a role of a cytosolic pathway in maintaining overall phenylalanine homeostasis in the cell, despite its relatively low direct contribution to phenylalanine biosynthesis. In addition, our results demonstrate that the phenylpyruvate route contributes to phenylacetaldehyde emission upon mechanical wounding in Arabidopsis (Fig. 4e). The experimental evidence that the use of multiple TSS is tissue specific and stress responsive^41,63^ and that cytosolic localization of PDT isoform is the result of transcription from an alternative TSS of a gene encoding a plastidial enzyme, suggests that the phenylpyruvate pathway likely contributes to phenylalanine biosynthesis when demand for phenylpropanoids is increased as a part of plant biotic and abiotic stress responses. Similarly, tyrosine biosynthesis via a cytosolic prephenate dehydrogenase in legumes has been attributed to demand of specialized lineage-specific metabolites^64^.

## Methods

### Generation of *PhCM2-*RNAi transgenic plants

*P. hybrida* cv Mitchell plants were grown under standard greenhouse conditions with a light period from 6:00 h to 21:00 h. For the *PhCM2*-RNAi construct, a fragment of *PhCM2* coding region (334 bp in size, corresponding to nucleotides 100 to 434) was amplified by PCR using forward 5’-CTCGAGTCTAGAATCAAATTCCCAATAAATTCCACC-3’ and reverse 5’-GAATTCGGATCCTGAATATCACAGGCAGCAGT-3’ primers. The PCR fragment was subcloned into the polylinker site (*Xho*I/*Eco*RI) upstream of the intron of a modified pRNA69 vector, containing the *Clarkia breweri linalool synthase* (*LIS*) petal-specific promoter^31^, and in the inverted orientation into the polylinker site (*Bam*HI/*Xba*I) downstream of the intron. The entire cassette containing the *LIS* promoter and the *PhCM2* hairpin structure was released by *Sac*I/*Not*I digestion and ligated into the corresponding sites of the pART27 binary vector^65^. The final *PhCM2* RNAi construct in the binary vector was used for *Agrobacterium tumefaciens* (GV3101)-mediated transformation of *P. hybrida* cv. Mitchell using the standard leaf disk transformation method.

### Isolation of homozygous Arabidopsis *AtCM2* mutants

*Arabidopsis thaliana AtCM2* T-DNA insertion lines N414383/*cm2-1* (Nottingham Arabidopsis Stock Centre, UK) and CS849985/*cm2-2* (Arabidopsis Biological Research Center, Ohio State University, USA) were grown under standard greenhouse conditions with a light period from 6:00 h to 22:00 h. Homozygous *cm2*-mutant plants were identified by PCR analysis on isolated genomic DNA using gene-specific primers flanking the T-DNA insertions (CM2-L/CM2-R) as well as T-DNA border-specific primers (o8409 and p745 for *cm2-1* and *cm2-2*, respectively) (Supplementary Table 1). Rosette leaves of 4-week-old wild-type and *cm2*-mutant plants were wounded using an array of 50 household needles, and volatiles were collected for 24 h^36^.

### qRT-PCR analysis and 5′-RLM-RACE

Sample collection, RNA isolation, and qRT-PCR were performed using established protocols^66^. Unless otherwise specified, tissues were harvested at 20:00 h. Briefly, total RNA was isolated using a Spectrum^TM^ Plant Total RNA Kit (Sigma, St. Louis, MO, USA) and treated with DNase I (Promega, Madison, WI, USA) to eliminate genomic DNA. One microgram of total RNA was reverse transcribed to cDNA using a 5X All-In-One RT MasterMix (Applied Biological Materials, Richmond, BC, Canada). Relative quantification of *AtAAS*, *AtCM2, AtADT6*, *PhCM1*, *PhCM2*, *PhADT1*, *PhADT2*, *PhADT3*, *DAHPS*, *EPSPS,* and *ODO1* transcript levels were performed by qRT-PCR analysis with gene-specific primers (Supplementary Table 1) relative to the reference genes *UBQ10* for petunia and *Ubc* for Arabidopsis^10,16,36^. Changes in *PhADT3S, PhADT3L,* and *PhCM2* transcript levels during a normal light/day cycle were analyzed by qRT-PCR using elongation factor 1-α (*EF1-α*) as a reference gene^67^. Each data point represents an average of three independent biological replicates, unless indicated. *AtCM2* transcript levels in wild type, *cm2-1*, and *cm2-2* were analyzed by RT-PCR^36^.

The 5′-RLM-RACE was performed using the GeneRacer^TM^ Kit (Invitrogen) according to the manufacturer’s instructions. Obtained PCR fragments were subcloned into pCR-TOPO vector (Invitrogen) and sequenced.

### Metabolic profiling

Volatiles were collected from detached 2-day-old flowers of control and transgenic lines from 18:00 to 22:00 h using the closed-loop stripping method^31^. Trapped volatiles were eluted from Porapak Q traps (80/100 mesh size; Waters, Milford, MA) with 300 μl dichloromethane containing naphthalene (200 μg/ml) as an internal standard. Samples were analyzed by gas chromatography (GC)-MS using 5975 inert XL EI/CI mass spectrometer detector combined with 6890N GC (Agilent Technologies, Santa Clara, CA) with an Agilent 19091S-433 HP-5MS capillary column (30 m × 0.25 mm; film thickness 0.25 μm)^10^. Then, 2 μl of sample was injected in pulsed splitless mode with an injector temperature of 250 °C. Column temperature was held at 40 °C for 3 min and then heated to 220 °C at 8 °C/min. Electron ionization energy was set at 70 eV. Mass spectra were obtained in scan mode scanning across 50 to 550 amu. All volatile compounds were identified by comparing their retention times and mass spectra with those of corresponding authentic compounds. Quantification was performed using calibration curves generated from individual authentic standards.

Internal pools of aromatic amino acids and organic acids (shikimate, prephenate, arogenate, and phenylpyruvate) from control and transgenic petals (minimum of six flowers per biological replicate) were extracted at 20:00 h on day 2 postanthesis and analyzed by LC-MS^10^. Each data point represents an average of six to nine independent biological replicates. For analysis of aromatic amino acids and arogenate, 0.2 g tissue was ground in liquid N_2_ and homogenized in 0.8 ml of 75% ethanol containing 0.5% 2-amino-2-methyl-1-propanol HCl (pH 10) with 30 nmol of α-aminoadipic acid as an internal standard. After centrifugation, 500 μl of the supernatant was filtered through an Amicon Ultra filter (Ultracel-10K, MilleporeSigma, Darmstadt, Germany), vacuum dried, and dissolved in 50 μl water. Then, 10 μl of sample was derivatized with *o*-phthaldialdehyde and injected into LC-MS. Chromatographic separations were performed using an Agilent 1100 HPLC system and a Luna C18 (2) column (3 μm, 100 Å, 250 × 4.6 mm; Phenomenex) with a 25 min linear gradient of 25–90% methanol in 0.1% ammonium acetate (v/v) at a 0.3 ml/min flow rate. The column effluent was then introduced by negative electrospray ionization (ESI) into an Agilent 6210 MSD time-of-flight mass spectrometer^10^. Compounds were quantified based on a calibration curves generated with authentic standards.

For analysis of shikimate, 0.5 g of tissue was ground in liquid N_2_ and homogenized in 1 ml of 0.25 N HCl. After 20 min of centrifugation, the supernatant was filtered through a 0.45 μm SFCA Nalgene syringe filter (Thermo Scientific) and analyzed by LC-MS as above using an Agilent ZORBAX SB-C18 column (4.6 × 150 mm × 3.5 μm) with an isocratic elution of 0.1% formic acid (v/v) in ddH_2_O at a 0.5 ml/min flow rate. Shikimic acid was quantified based on a calibration curve generated with authentic standard.

For prephenate and phenylpyruvate, tissue extracts were prepared as described above for aromatic amino acids with the exception of using 4-chlorobenzoic acid as an internal standard. For prephenate detection, extract was prepared at pH 10 to degrade endogenous phenylpyruvate and subsequently acidified by 0.25 N HCl for 1 h at 37 °C to completely convert endogenous prephenate to phenylpyruvate^10^. For phenylpyruvate detection, petal extracts were prepared at pH 6, in which phenylpyruvate peak represented a sum of endogenous phenylpyruvate and the phenylpyruvate produced by acid conversion of prephenate during extraction. The difference between phenylpyruvate levels in the petal extracts prepared at pH 6 and pH 10, the latter treated with HCl, was used to determine endogenous phenylpyruvate content. Then, 10 μl of the sample was analyzed by LC-MS as above using an Agilent ZORBAX SB-C18 column (4.6 × 150 mm × 3.5 μm) with a 10 min linear gradient from 20 to 70% methanol in 0.1% ammonium acetate (v/v) at a 0.5 ml/min flow rate. Peak quantification was performed based on a calibration curve generated from the authentic standard.

### Yeast complementation assays

Coding regions of *PhADT1*, *PhADT2*, and *PhADT3* were first amplified using gene-specific primers (Supplementary Table 1) possessing 5′-extensions that provide annealing sites for subsequent amplification with AttB primers (Invitrogen). The resulting amplicons were inserted into pDONR/Zeo vector by recombination using BP Clonase II (Invitrogen) according to the manufacturer’s protocol. The generated constructs were then subjected to recombination with the yeast expression vector pYES-DEST52 using LR Clonase II (Invitrogen) following the manufacturer’s protocol. The *S. cerevisiae PHA2* open reading frame was acquired in a yeast expression vector to be used as a positive complementation control (Dharmacon, Lafayette, CO), while pYES-DEST52-*Gus*, generated by LR recombination as above with pENTR-Gus vector, was used as a negative control. Yeast expression constructs were transformed into haploid *pha2* knockout *S. cerevisiae* derived from BY4742 strain (Matα, *his*3∆1, *leu*2∆0, *lys*2∆0, *ura*3∆0, YNL316c:kanMX4; Dharmacon, Lafayette, CO) using the Frozen-EZ Yeast Transformation II kit (Zymo Research, Irving, CA). To test for complementation, yeast were grown overnight at 30 °C in synthetic defined uracil dropout media containing 2% raffinose. Resulting cultures were diluted to OD_600_ of 0.05 in synthetic defined phenylalanine dropout media containing 2% galactose, and continued to grow at 30 °C. Yeast growth was monitored by absorbance at 600 nm every 24 h.

### Enzyme assays

For biochemical characterization of mature PhCM1 (minus predicted transit peptide) and PhCM2 as well as mature PhADT3L and PhADT3S, the coding region of the corresponding gene was amplified using forward and reverse primers (Supplementary Table 1) containing *Nde*I and *Bam*HI restriction sites, respectively, for directional cloning into pET-28a expression vector (Novagen, Madison, WI) in-frame with an N-terminal 6XHis-tag. After sequence verification, recombinant proteins were produced in *E. coli* and purified on Ni-NTA resin (Qiagen, Hilden, Germany)^10^. Since *E. coli* crude extracts contain endogenous CM and PDT activities derived from a bifunctional CM/PDT enzyme, CM activity was monitored in purified PhADT3L and PhADT3S fractions, while PDT activity was checked in purified PhCM1 and PhCM2 fractions to ensure the absence of *E. coli* CM/PDT contamination.

CM and PDT activity assays of recombinant proteins were carried out in 50 μl reaction mixture containing 20 mM Tris-HCl (pH 8.0), 1 mM EDTA, 500 μM chorismate or prephenate and 0.3–3 ng (for CM) and 2 μg (for PDT) recombinant enzyme, unless otherwise noted. The reaction mixtures were incubated at 37 °C for 20 min and 30 min for CM and PDT activities, respectively. For CM activity, reaction was terminated by adding 50 μl 1 N HCl followed by incubation at 37 °C for 20 min to convert prephenate into phenylpyruvate. Then, 150 μl of 2.5 N NaOH was added before measuring phenylpyruvate formation by spectrophotometer at 320 nm. Due to the need for higher sensitivity at low substrate concentrations, assays with recombinant PhCM2 were performed as above, except that reaction volume was increased to 225 μl, and 10 μl of 10 N HCl was added for the conversion of prephenate to phenylpyruvate, while15 μl of 25 N NaOH was added prior to the spectrophotometric analysis. For PDT activity, the reaction was stopped with 200 μl of 2.5 N NaOH and phenylpyruvate formation was measured by spectrophotometer at 320 nm and confirmed by LC-MS.

Chorismate was prepared using *Klebsiella pneumonia*, strain 62-1 (American Type Culture Collection, Manassas, Virginia), in which consumption of chorismate is drastically decreased leading to its secretion to the growth media^68,69^. Briefly, 1 l of sterile growth media (per liter: 2 g yeast extract, 2 g casein hydrolysate, 41 mg tryptophan, 2 g citric acid, 0.2 g anhydrous magnesium sulfate, 13.5 g dibasic sodium phosphate, 1 g monobasic potassium phosphate, and 2 g ammonium chloride) was inoculated with 1 ml of overnight LB culture of *K. pneumonia* and grown with shaking for approximately 6 h at 30 °C until OD_625_ reached 1.8. Cells were pelleted by centrifugation at 5000 × *g* for 5 min, the supernatant was discarded, and cells were resuspended in 1 l freshly prepared accumulation media (per liter: 10 g glucose, 19.2 g dibasic sodium phosphate, 2.05 g monobasic potassium phosphate, 2.7 g ammonium chloride, 21 mg magnesium chloride septa-hydrate, and 3 mg tryptophan). Cells were grown for 20 h with shaking at 30 °C, then pelleted by centrifugation at 5000 × *g* for 5 min and the supernatant was used for chorismate purification. After increasing pH to 8.5, the supernatant was loaded under vacuum to the prepared DOWEX column with pH adjusted below 9.0 (30 g of DOWEX-Cl 1 × 8, 200 mesh, Sigma-Aldrich). After washing with 80 ml of water, chorismate was eluted with 2 M ammonium chloride (pH 8.5). Chorismate-containing fractions (6 ml each) were identified by absorption at 275 nm, pooled together and pH was adjusted to 1.5 by HCl. Non-polar contaminants were removed via partitioning with 75 ml dichloromethane three times. The final aqueous phase was extracted three times with 50 ml ethyl acetate to recover the chorismate. The ethyl acetate fractions were combined, dried with anhydrous sodium sulfate, and evaporated under vacuum. The resulting residue was dissolved in 5 ml of 10 mM ammonium acetate (pH 6.0), applied to a 1.5 × 20 cm column packed with 25 g octadecyl-functionalized silica gel (Sigma) that was pre-equilibrated with 250 ml 10 mM ammonium acetate (pH 6.0). Column was eluted with 10 mM ammonium acetate (pH 6.0) at a flow rate of 10 ml/min, and 4 ml fractions were collected. Fractions containing chorismate were pooled and lyophilized. The resulting chorismate powder was resuspended in water, the concentration was determined by absorption at 275 nm (ε = 2630), and aliquots stored at −80 °C until needed. Chorismate was assessed by nuclear magnetic resonance and purity was estimated as >90% by LC-MS.

Prephenate was enzymatically synthesized from chorismate using PhCM2 and purified following an established protocol^70^. Following enzymatic conversion of chorismate to prephenate, the reaction mixture was diluted with water to an approximate prephenate concentration of 20 mM. To each 10 ml of prephenate solution, 3.3 ml of ice-cold BaBr_2_ solution (1.4% barium bromide in 80% ethanol) was added, and the mixture was incubated for 1 h on ice. Following centrifugation at 13,000 × *g* for 30 min at 4 °C, the resulting supernatant was mixed with BaBr_2_ solution at 2:1 ratio and immediately ethanol was added to final concentration of 85%. The resulting solution was incubated on ice for 16 h, then centrifuged at 13,000 × *g* for 30 min at 4 °C and the supernatant discarded. The pellet was washed twice with 100% ethanol, then twice with ethyl ether, and centrifuged at 13,000 × *g* for 10 min at 4 °C after each solvent wash. The final pellet was dried by overnight lyophilization, and the resulting barium salt of prephenic acid was dissolved in water. The concentration was determined by absorption at 320 nm after the conversion of prephenate to phenylpyruvate by acid hydrolysis as described above.

All enzyme assays were performed at an appropriate enzyme concentration so that reaction velocity was proportional to enzyme concentration and linear during the incubation time period. Kinetic data were evaluated by nonlinear regression analysis (GraphPad Prism, version 6.05). Triplicate assays were performed for all data points except as otherwise specified.

### Preparation of plastidial and cytosolic fractions

Plastidial and cytosolic fractions were prepared from 1- to 3-day-old petunia petals harvested at 10:00 h, using established protocol^16^ with some modifications. A total of 20 g of petal tissue was submerged in 200 ml chilled homogenizing buffer A containing 0.5 M sorbitol, 20 mM HEPES/NaOH (pH 7.4), 10 mM KCl, 1 mM MgCl_2_.6H_2_O, 1 mM Na_2_-EDTA, 5 mM EDTA, and 5 mM dithiothreitol (DTT) and blended three times for 2 s. The homogenate was filtered through two layers of Miracloth (Calbiochem, La Jolla, CA, USA). A 1 ml aliquot was removed to analyze enzyme activities in crude extracts, and the remainder was centrifuged at 2500 × *g* for 10 min. The pellet was washed and resuspended in 2 ml of buffer B containing 0.66 M sorbitol, 20 mM HEPES/NaOH (pH 7.5), and layered over a Percoll^TM^ gradient consisting of 2 ml 80% and 5 ml 30% Percoll^TM^ in buffer B. Intact plastids were collected from the interface of 80 and 30% Percoll^TM^ after centrifugation at 5000 × *g* for 20 min at 4 °C, with both slow acceleration and deceleration settings. Plastids were washed in buffer B and resuspended in 50 mM Na-phosphate (pH 8.0) and 10% glycerol buffer. For a cytosolic fraction, the remaining supernatant was centrifuged at 12,000 × *g* for an additional 30 min at 4 °C to eliminate mitochondria and peroxisomes. The resulting supernatant was then desalted through Sephadex G-50 columns (GE Heathcare) and eluted into 50 mM Na-phosphate (pH 8.0) and 10% glycerol buffer and concentrated using Amicon® Ultra -10K centrifugal filters (EMD Millipore, Billerica, MA, USA). Obtained fractions as well as crude protein extracts were used for analysis of PPA-AT, alcohol dehydrogenase ADH (cytosolic marker), and CM activities.

For detection of PPA-AT activity, assays were conducted in 50 μl of reaction mixture containing 50 mM sodium phosphate buffer (pH 8.0), 20 mM L-aspartate, 200 μM PLP, 2 mM prephenate, and 1 to 3 μg of total protein. After incubation at 37 °C for 15 min, the reaction was terminated by 25 μl of 1 N HCl and further incubated at 37 °C for 20 min for quantitative conversion of arogenate to phenylalanine^21^ before neutralization with 25 μl of 1 N NaOH. Then, 10 μl of the final reaction mixture was derivatized with *o*-phthalaldehyde (OPA) and subjected to high-performance liquid chromatography (HPLC) analysis using an Agilent 1200 HPLC system (Palo Alto, CA) equipped with ZORBAX Eclipse XDB-C18 column (5 μm, 80 Å, 150 × 3 mm; Agilent) with a 15 min linear gradient of 15 to 65% methanol in 20 mM sodium phosphate buffer (pH 6.8). Phenylalanine production was monitored at 336 nm and quantified based on a standard calibration curve generated with authentic standard.

ADH activity assays were conducted in 500 μl of reaction mixture containing 100 mM sodium phosphate buffer (pH 8.0), 1 mM NAD^+^, 100 μM ethanol, and 1 to 3 μg of total protein. NADH production was measured spectrophotometrically at 340 nm over 5 min of incubation at 25 °C, and quantified based on a standard calibration curve.

ADT activity in crude extracts was analyzed in 20 μl reaction mixture containing 500 μM arogenate, 250 mM sodium phosphate buffer (pH 8.2), 1 mM EDTA, and 1 to 3 μg of total protein. Mixture was incubated at 37 °C for 15 min and reaction was terminated by adding 25 μl of methanol. Produced phenylalanine was quantified as described above.

### Non-aqueous fractionation and enzyme assays

To independently evaluate subcellular distribution of phenylalanine biosynthetic enzymes, petal tissue of 2-day-old wild-type flowers harvested at 21:00 h was subjected to non-aqueous fractionation^32^. Approximately 0.3 g of dried tissue was resuspended in 2.5 ml heptane/tetrachloroethylene (density = 1.32 g/ml) and homogenized in a ball mill. The resulting suspension was layered atop a freshly prepared heptane/tetrachloroethylene density gradient, then centrifuged for 90 min at 13,000 × *g*, 4 °C. The resolved gradient was divided into six fractions and all solvent evaporated under nitrogen flow. The resulting fraction residues were resuspended in 1 ml of protein resuspension buffer (50 mM Tricine-NaOH, 10% glycerol, pH 8.4), centrifuged, and metabolites were removed using Zeba^TM^ Spin Desalting Columns (7 K MWCO, Thermo Scientific). The resulting fractions were first assayed for marker activities: NADP-GAPDH for plastids and phospho*enol*pyruvate (PEP) carboxylase for cytosol. Then, activities of PAL, a well-known cytosolic enzyme, and PPA-AT, a plastidial enzyme, were used to validate the subcellular resolution. Finally, CM assays were performed across a gradient in the presence of 20 μM and 500 μM of chorismate as well as PDT activities were analyzed as described above. PPY-AT activity was analyzed in 50 μl reaction mixture containing 50 mM HEPES, pH 10, 10 mM tyrosine, 20 mM phenylpyruvate, and 200 mM PLP. The reaction was initiated by adding 10 μl of fractionated protein and incubated at 30 °C for 20 min. After termination of reaction with 50 μl of 1 N HCl and centrifugation, 10 μl of the final reaction mixture was derivatized with OPA and phenylalanine production was analyzed by HPLC as described above.

For GAPDH activity assays^71^, 37.5 μl of fractionated protein was incubated in 50 μl activation mixture containing 10 mM DTT, 5 mM ATP, 20 mM sodium phosphate buffer (pH 8.4), and 50 mM Tricine-NaOH (pH 8.4). After 10 min of incubation at room temperature, 200 μl of reaction mixture (50 mM Tricine-NaOH, pH 8.4, 25 mM MgCl_2_, 10 mM DTT, 5 mM ATP, 50 μM NADPH, 10 mM 3-phosphoglycerate, 3 units of phosphoglycerate kinase) was added. NADPH consumption was measured spectrophotometrically at 340 nm (ε = 6200 M^−1^ cm^−1^) over 5 min of incubation at 25 °C.

PEP carboxylase assays essentially followed the protocol described in ref. ^72^ using a 250 μl volume containing 50 mM Tricine-NaOH (pH 8.4), 5 mM MgCl_2_, 5 mM DTT, 10 mM NaHCO_3_, 2.5 mM PEP, 0.2 mM NADH, 2 units of malate dehydrogenase, and 20 μl of fractionated protein. For PAL activity assays^73^, reaction mixture contained 5 μl of fractionated protein and 2 mM L- phenylalanine in 50 μl assay buffer (100 mM Na_2_B_4_O_7_, pH 8.8, and 2 mM DTT). After incubation for 30 min at 30 °C, the reaction was stopped with 25 μl of 1 N HCl and conversion of phenylalanine to *trans*-cinnamic acid was measured by HPLC. Distribution of enzymatic activities between compartments was solved by best fit algorithm^38^.

### Shikimate feeding

The 2-day-old *PhCM2*-RNAi and control petunia petals were fed with 100 mM shikimate for 7 h (from 15:00 h to 22:00 h). Levels of phenylalanine, tyrosine, tryptophan, arogenate, prephenate, and phenylpyruvate were analyzed by LC-MS as described above.

### Metabolic flux analysis with ^15^N-tyrosine labeling

The 2-day-old *PhCM2*-RNAi and control petunia petals were fed with 10 mM ^15^N-tyrosine (Cambridge Isotope Laboratories) starting at 18:00 h. After 2 h, 4 h, and 6 h of feeding, the isotopic abundances and pool sizes of phenylalanine, tyrosine, and arogenate were analyzed by LC-MS and used for metabolic flux analysis^16,32^. No labeling of arogenate was detected at any timepoint.

### PhADT3 immunodetection and proteomic analyses

PhADT3 was immunoprecipitated from 0.5 g of petals of 2-day-old wild-type flowers harvested at 10:00 h using rabbit anti-PhADT3 antibodies and Pierce™ Classic IP Kit (Thermo Scientific) according to the manufacturer’s protocol. Rabbit anti-PhADT3 polyclonal antibodies were generated against a synthetic PhADT3 peptide CFKTSIVFAHDKGTS and purified by cross-absorption with PhADT1-specific peptide CFKTSIVFAHEGTGV to increase antibodies specificity and prevent cross-reactivity with PhADT1 (Genscript, Piscataway, NJ). Then, 1 mg of total protein crude extract was incubated with 1.5 µg of anti-PhADT3 polyclonal antibodies at 4 °C overnight. The PhADT3 immune complex was recovered in 100 µl of elution buffer and was subjected to western blot and proteomic analyses.

Western blot analysis of isolated PhADT3 immune complex (4 µl) was carried out as previously described^74^. Immunodetection was performed on 0.06 µg of protein using rabbit anti-PhADT3 antibodies (1:5000 dilution) and Pierce™ goat anti-rabbit IgG (H+L) horseradish peroxidase conjugate (1:10,000 dilution) as secondary antibodies (Catalog no. 31460, Thermo Scientific). Antigen bands were visualized using chemiluminescent substrate for detection of horseradish peroxidase (Pierce™ ECL Western Blotting Substrate, Thermo Scientific) according to the manufacturer’s protocol and exposing the membranes on UltraCruz® Autoradiography Film (Santa Cruz Biotechnology). Purified recombinant mature PhADT3L (~42 kD), PhADT3S (~33 kD), and PhADT1 proteins as well as preparations from *E. coli* carrying empty pET28 expression vector were used as controls.

A total of 60 µl of isolated PhADT3 immune complex prepared from crude extract were loaded on a gel and used for proteomics analysis. Approximately 0.3 cm sections were excised from SDS-PAGE gels from positions corresponding to the bands detected in immunoblots from identical gels with anti-PhADT3 antibodies. Gel sections were destained and digested with trypsin (Sequencing grade, Promega) overnight^75^ and analyzed at the Proteomics Facilities (Bindley Bioscience Center, Purdue University) as follows. Samples were subjected to reverse-phase HPLC-ESI-MS/MS using the Dionex UltiMate 3000 RSLC nano System (Thermo Fisher Scientific) coupled to the Q-Exactive High Field (HF) Hybrid Quadrupole Orbitrap MS (Thermo Fisher Scientific) and a Nano-electrospray Flex ion source (Thermo Fisher Scientific). Purified peptides were loaded onto a trap column (300 µm ID × 5 mm) packed with 5 µm 100 Å PepMap C18 medium and washed using a flow rate of 5 µl/min with 98% purified water/2% acetonitrile (ACN)/0.01% formic acid (FA). After 5 min, the trap column was switched in-line with the analytical column. Peptides were separated using a reverse-phase Acclaim PepMap RSLC C18 (75 µm × 15 cm) analytical column using a 120 min method at a flow rate of 300 nl/min. The analytical column was packed with 2 µm 100 Å PepMap C18 medium (Thermo Scientific). Mobile phase A consisted of 0.01% FA in water and a mobile phase B consisted of 0.01 % FA in 80% ACN. The linear gradient started at 5% B and reached 30% B in 80 min, 45% B in 91 min, and 100% B in 93 min. The column was held at 100% B for the next 5 min before being brought back to 5% B and held for 20 min. Sample was injected into the QE HF through the Nanospray Flex™ Ion Source fitted with an emission tip from Thermo Scientific. Column temperature was maintained at 35 °C. MS data were acquired with a Top20 data-dependent MS/MS scan method. The full scan MS spectra were collected over 300–1650 *m/z* range with a maximum injection time of 100 ms, a resolution of 120,000 at 200 *m/z*, spray voltage of 2, and AGC target of 1 × 10^6^. Fragmentation of precursor ions was performed by high-energy C-trap dissociation with the normalized collision energy of 27 eV. MS/MS scans were acquired at a resolution of 30,000 at *m/z*. The dynamic exclusion was set at 20 s to avoid repeated scanning of identical peptides. Instrument optimization and recalibration was carried out at the start of each batch run using Pierce calibration solution. The sensitivity of the instrument was also monitored using *E. coli* digest at the start of the sample run.

LC-MS/MS data were searched using MaxQuant software (v. 1.5.3.28)^76–78^ with its built-in Andromeda search engine^76^ for protein identification and label free MS1 quantitation. The MS/MS spectra were searched against the *Solanum lycopersicum* protein database downloaded from the NCBI (National Center for Biotechnology Information). The minimal length of six amino acids was required in the database search. The database search was performed with the precursor mass tolerance set to 10 ppm and MS/MS fragment ions tolerance was set to 20 ppm. Database search was performed with enzyme specificity for trypsin/LysC, allowing up to two missed cleavages. Oxidation of methionine was defined as a variable modification, and carbamidomethylation of cysteine was defined as a fixed modification. The ‘unique plus razor peptides’ were used for peptide quantitation. Razor peptides are the non-unique peptides assigned to the protein group with the most other peptides. The false discovery rate of peptides and proteins identification was set at 1%. At this stringency, only one peptide corresponding to known ADT enzymes was identified. This peptide (LVDDANVGTAK) was common to both PhADT1 and PhADT3 at a position downstream of the second methionine, and was present in both the high and low molecular weight bands. To verify the presence of the target protein, a direct database search was performed as above using PhADT3 as query, which led to the identification of the three peptides AVLPIENSLGGSIHR, NYDLLLR, and VAYQGVPGAYSEAAAGK, the first of which is exclusive to PhADT3 among petunia ADT proteins.

### Subcellular localization of PhADT3S

The coding region of PhADT3S was amplified both with and without the native stop codon using gene-specific primers (Supplementary Table 1) possessing 5′-extensions that provide annealing sites for subsequent amplification with AttB primers (Invitrogen). The resulting amplicons were inserted into pDONR/Zeo vector by recombination using BP Clonase II (Invitrogen) according to the manufacturer’s protocol. The generated constructs with and without the stop codon were then subjected to recombination with binary GFP-fusion vectors pK7WGF2 (N-terminal GFP) and pK7FWG2 (C-terminal GFP), respectively, both containing the CaMV 35S promoter^79^, using LR Clonase II (Invitrogen) according to the manufacturer’s protocol. The constructs and a GFP cytosolic control were transformed into *Agrobacterium tumefaciens* (GV3101) and infiltrated into *Nicotiana benthamiana* leaves^80^. Five days after infiltration, leaf tissue was imaged using Zeiss LSM710 laser spectral scanning confocal microscope with a C-Apochromat 40 ×/1.20 W objective (Zeiss, Thornwood, NY, USA). GFP was excited with an Argon laser at wavelength 488 nm and emissions were collected over a 493–598 nm bandpass^16^. Chlorophyll fluorescence was excited by a HeNe laser at wavelength 633 nm and emissions were collected over a 647–721 nm bandpass^16^.

### *PhpCAT* overexpression and *PhADT3* downregulation in planta

Transient *PhpCAT* overexpression in wild-type and *PhCM2* RNAi petunia flowers was performed using previously generated pART27-derived *PhpCAT* overexpression construct^32^. Transient *PhpCAT* overexpression^16^ was achieved using vacuum infiltration of at least 20 total 1-day-old petunia flowers from each genetic background with *Agrobacterium tumefaciens* strain GV3101 containing pART27-LIS-*PhpCAT* and pART27-LIS vector (control) at OD_600_ of 0.4. At 48 h after infiltration, scent was collected for 6 h from 18:00 h to 24:00 h and analyzed by GC-MS. Immediately following scent collection, petal tissue was used for RNA analysis and metabolic profiling as described above.

For *PhADT3* RNAi downregulation, DNA containing two spliced *PhADT3* cDNA fragments of the coding region corresponding to nucleotides 2 to 549 and 2 to 340, the latter in antisense orientation to create a hairpin structure, was synthesized with flanking AttL1 and AttL2 recombination sites (Genscript, NJ). The resulting cassette was subcloned into the binary vector pB2GW7 (Invitrogen, Carlsbad, CA, USA) under control of the cauliflower mosaic virus 35S promoter using the Gateway LR Clonase II (Invitrogen) and used for transient downregulation as described above.

### Reporting summary

Further information on experimental design is available in the Nature Research Reporting Summary linked to this article.

## Supplementary information

Supplementary Information

Source Data

Reporting Summary

## Data Availability

Source data underlying figures are provided as a Source Data file. Proteomic raw data and MaxQuant search results have been made publicly available through MassIVE with submission ID: MSV000083169. Correspondence and requests for materials and raw data should be addressed to N.D. The computer code to evaluate the cytosolic and plastidial fluxes from the ^15^N labeling experiments are available from J.A.M.
