# Supplementary material for: Completion of the cytosolic post-chorismate phenylalanine biosynthetic pathway in plants
Source: Nat Commun. 2019 Jan 3;10:15. doi: 10.1038/s41467-018-07969-2 (PMC6318282; doi:10.1038/s41467-018-07969-2)
Supplement: Supplementary file 1 — Supplementary Information [file 41467_2018_7969_MOESM1_ESM.pdf]

Completion of the cytosolic post-chorismate phenylalanine biosynthetic pathway in plants.

Yichun Qian *et al.*

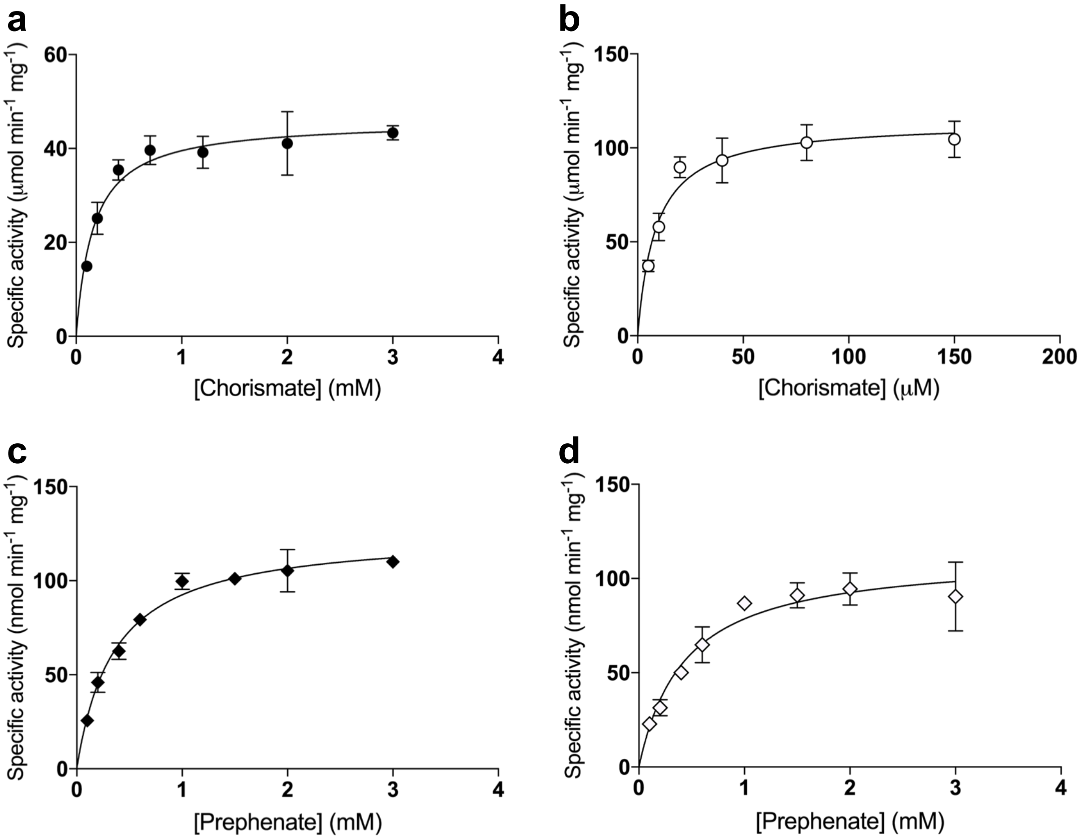

Supplementary Figure 1 | Michaelis-Menten kinetics for PhCM1 (a), PhCM2 (b), PhADT3L (c) and PhADT3S (d). Data are means ± SE (n=3).

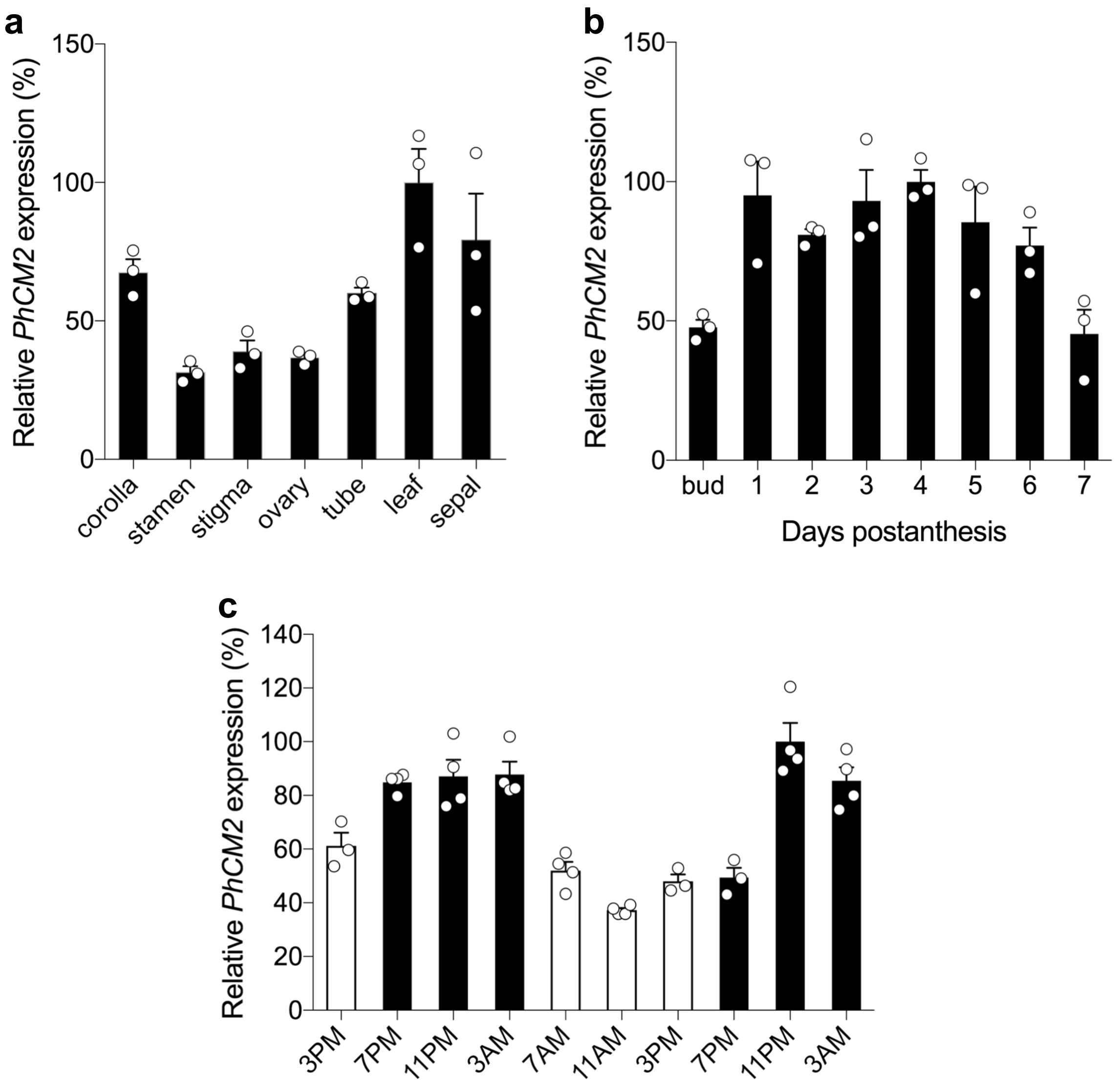

Supplementary Figure 2 | Expression profiles of *PhCM2* in petunia flowers.
(**a**) Tissue-specific expression of *PhCM2* presented relative to the level in leaf tissue set as 100%. (**b**) Developmental *PhCM2* expression profile in petunia corolla from mature buds to day 7 post-anthesis determined by qRT-PCR. Data are presented relative to the 4-day level. (**c**) Changes in *PhCM2* transcript levels during a normal light/day cycle in petunia corolla harvested at 15:00 h on day 1 to 03:00 h on day 3 post-anthesis. Black and white bars correspond to night and day sampling points, respectively. Data are presented relative to the transcript level at 23:00 h on day 2 post-anthesis set as 100%. All data are means ± SE (n = 3 biological replicates).

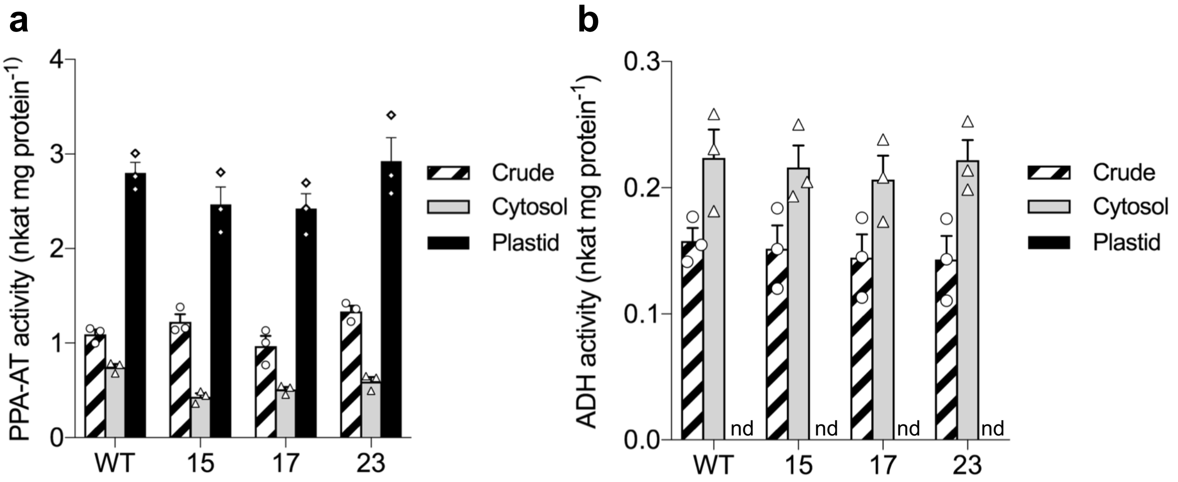

Supplementary Figure 3 | Activities of marker enzymes in WT and *PhCM2* RNAi petunia petals.

Prephenate aminotransferase (PPA-AT) (**a**) and alcohol dehydrogenase (ADH) (**b**) activities were used as a plastidial and cytosolic marker, respectively. Enzyme activities were measured in crude extracts (striped bars), cytosolic (grey bars) and plastidic (black bars) fractions prepared from corollas of 1 to 3 day-old wild type and *PhCM2* RNAi petunia flowers (lines 15, 17, and 23) harvested at 10:00 h. Data are means ± SE. (n=3 biological replicates). nd, not detected.

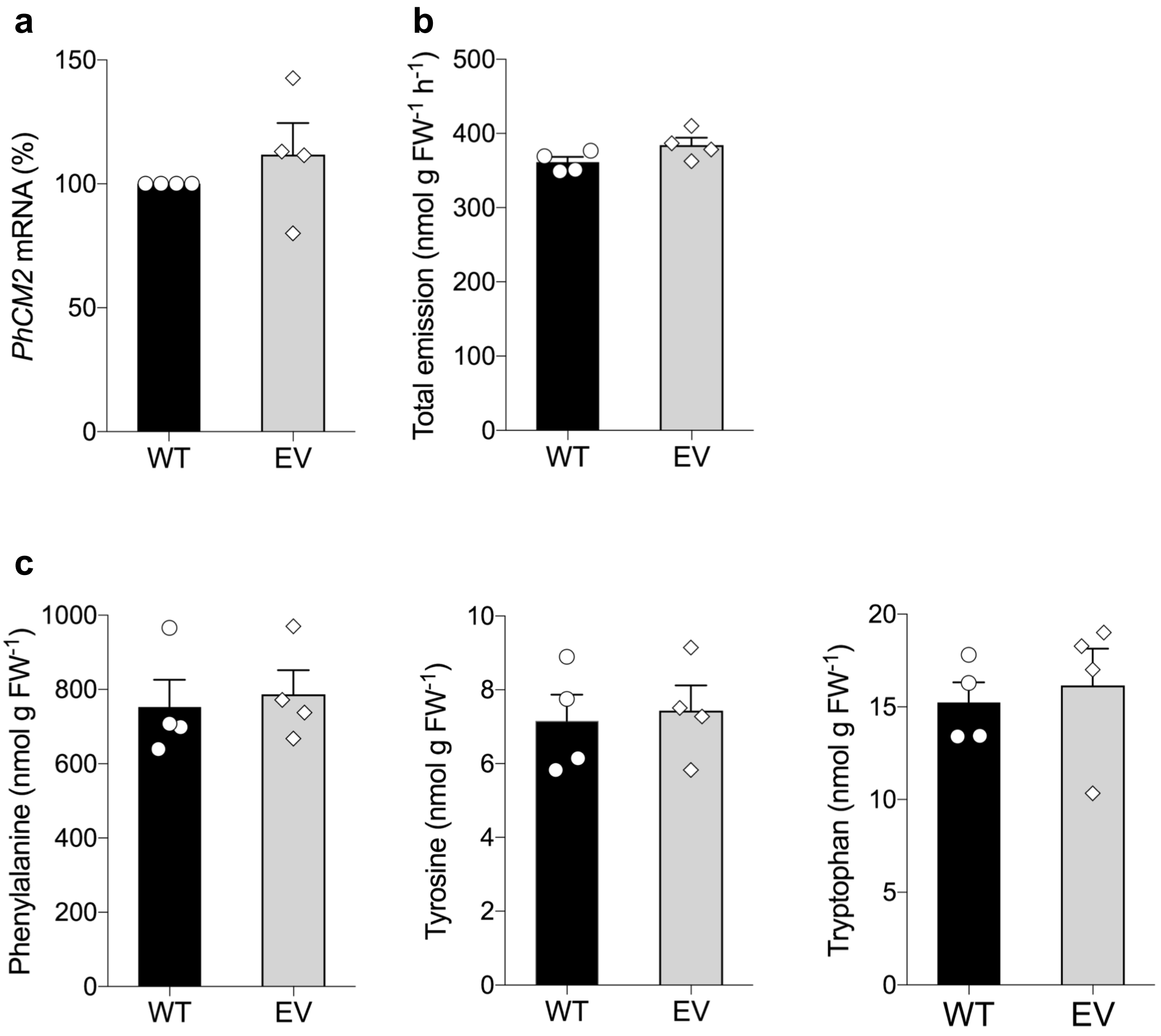

Supplementary Figure 4 | Levels of *PhCM2* transcripts (a), emitted phenylalanine-derived volatiles (b) and internal pools of aromatic amino acids (c) in petunia flowers of wild type (WT) and empty vector (EV) control lines.
Petal tissue for RNA isolation and aromatic amino acid extraction was harvested at 20:00 h on 2 day post-anthesis. Volatiles were collected from 2 day-old wild type (black bars) and transgenic (grey bars) petunia flowers expressing empty vector from 18:00 h to 22:00 h. *PhCM2* mRNA levels are presented as percentage of levels in wild-type set at 100%. Data are means ± SE (n = 4 biological replicates).

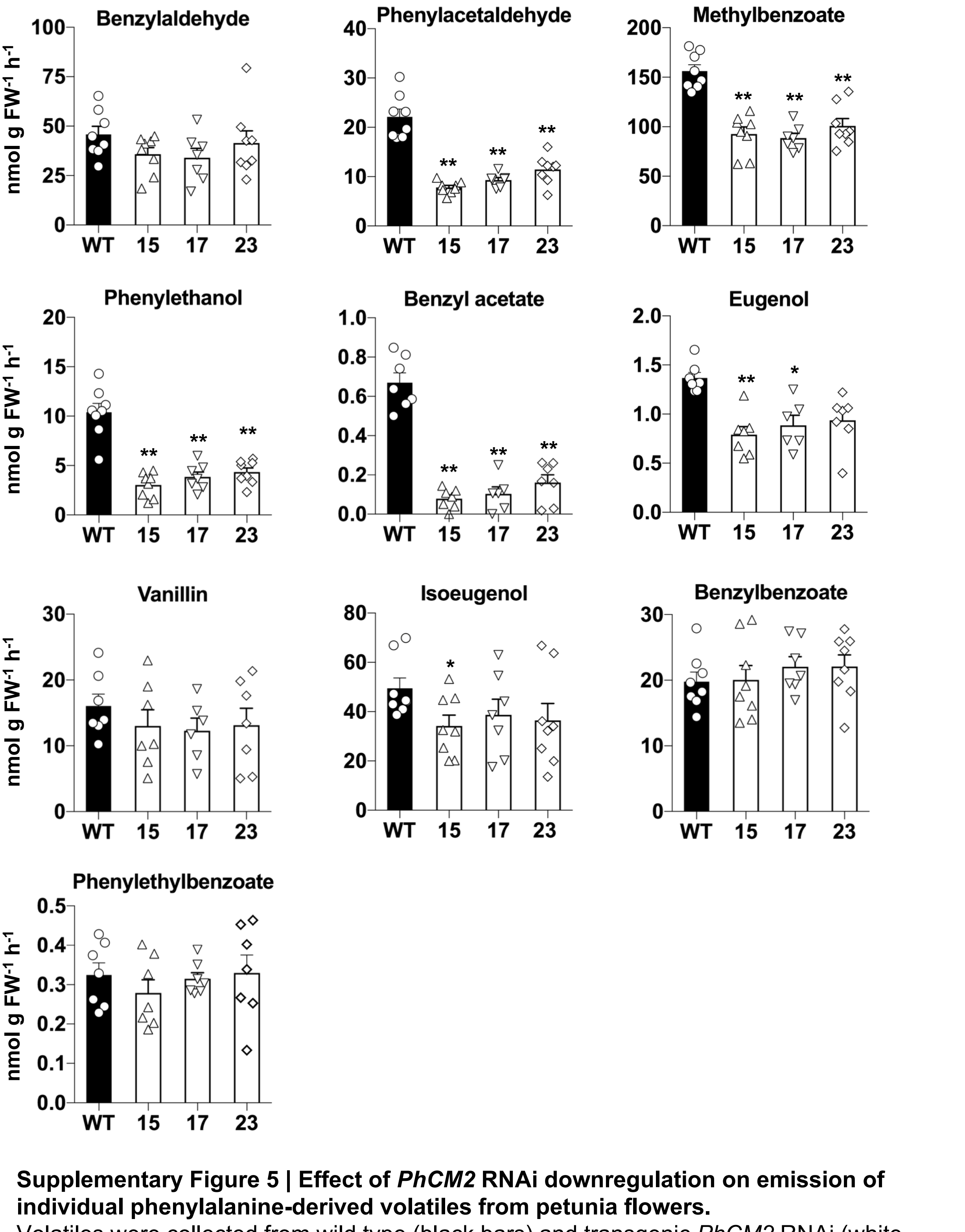

Supplementary Figure 5 | Effect of *PhCM2* RNAi downregulation on emission of individual phenylalanine-derived volatiles from petunia flowers.
Volatiles were collected from wild type (black bars) and transgenic *PhCM2* RNAi (white bars) petunia flowers from 18:00 h to 22:00 h on day 2 post-anthesis. Data are means ± SE (n ≥ 6 biological replicates). * $P < 0.05$, ** $P < 0.01$ as determined by paired two-tailed Student's *t*-test.

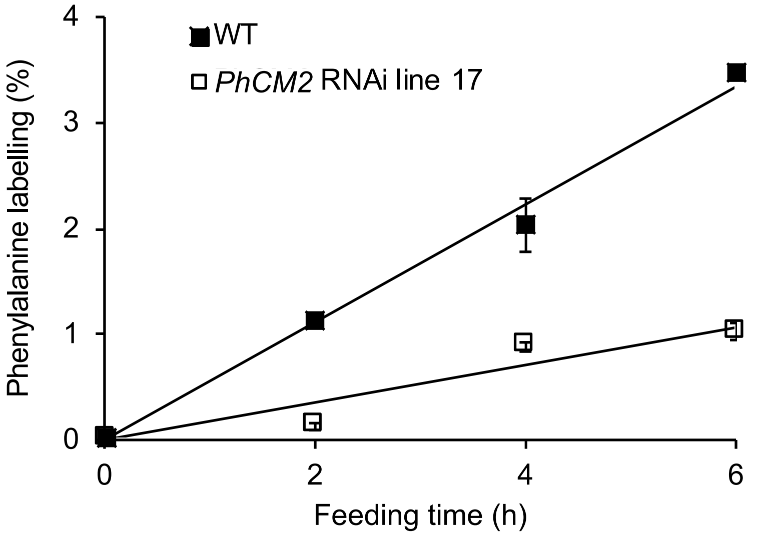

Supplementary Figure 6 | Isotopic labeling of phenylalanine from ^{15}N-tyrosine in petunia flowers.
10mM ^{15}N-Tyrosine was fed to 2 day-old control (solid square), and the *PhCM2* RNAi line 17 (open square) petunia flowers for 2, 4, 6 h beginning at 18:00 h. Labeling of phenylalanine pools were analyzed by TOF LC-MS. Data are means ± SE (n = 3 biological replicates). Incorporation of ^{15}N label in phenylalanine was linear over the 6-h time course (R^2 = 0.991 and 0.968 for control and the *PhCM2* RNAi line, respectively).

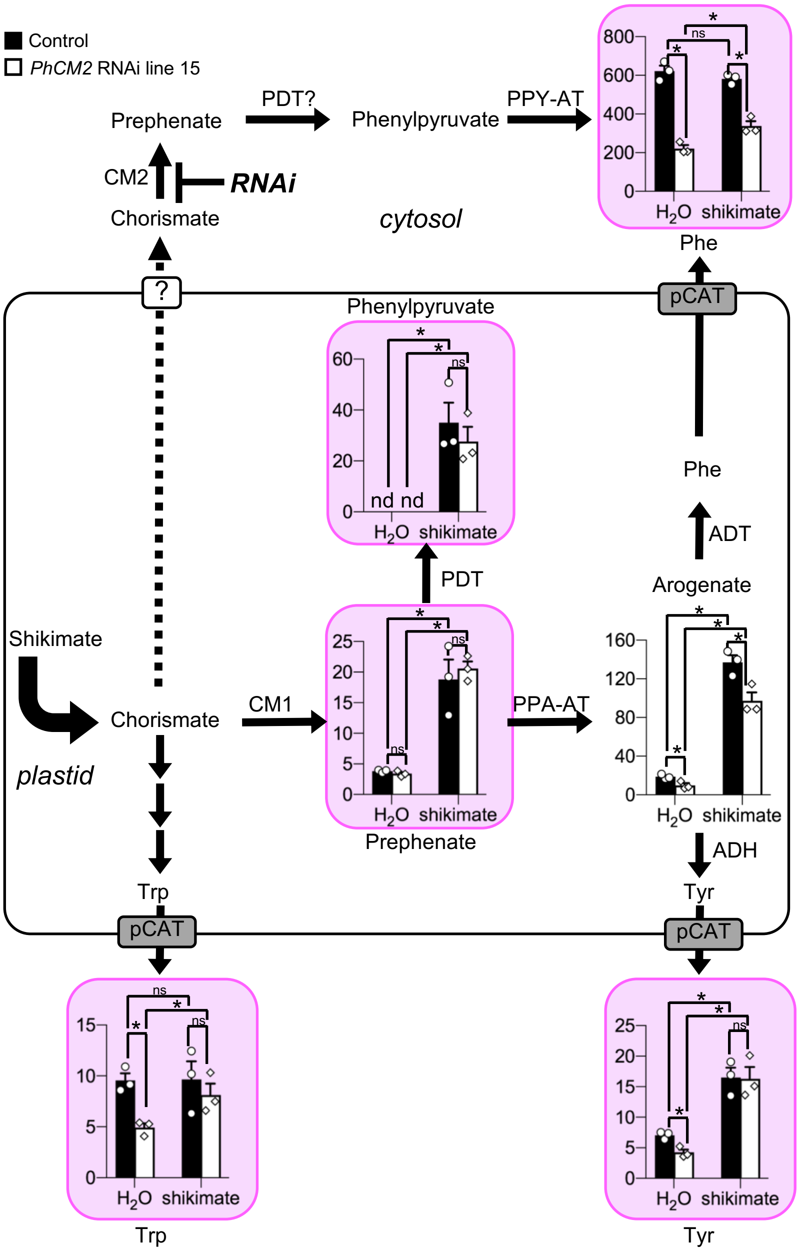

Supplementary Figure 7 | Effects of feeding with exogenous shikimate on the levels of aromatic amino acids, arogenate, prephenate and phenylpyruvate in wild type and *PhCM2* RNAi petunia petals.

Excised petals of wild type and *PhCM2* RNAi line 15 (black and white bars, respectively) were fed with water and 100 mM shikimate for 7 h (from 15:00 h till 22:00 h), and the levels of phenylalanine, tyrosine, tryptophan, arogenate, prephenate, and phenylpyruvate were analyzed. Metabolite levels are shown in nmol g FW^{-1}. Pink background indicates metabolites with potential dual subcellular localization, in the cytosol and plastids. Data are means ± SE (n = 3 biological replicates). * $P < 0.05$ as determined by paired two-tailed Student's *t* test. nd, not detected; ns, not significant.

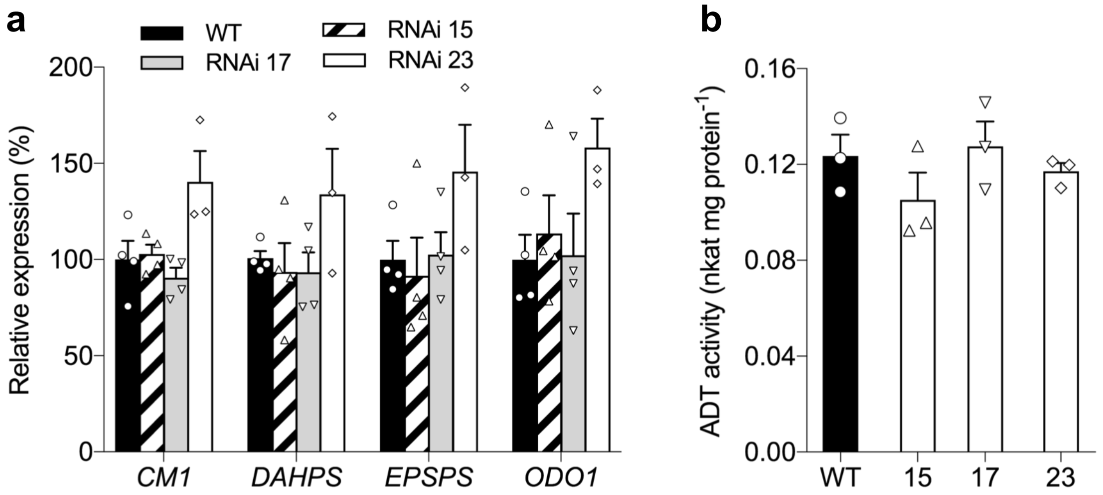

Supplementary Figure 8 | Effect of *PhCM2* downregulation on expression levels of shikimate pathway genes and ODO1 transcription factor, and plastidial ADT activity.

(**a**) Expression levels of *CM1,* 3-deoxy-D-arabino-heptulosonate 7-phosphate synthase (DAHPS), and 5-enolpyruvylshikimate 3-phosphate synthase (*EPSPS*), and their transcriptional activator ODORANT1 (*ODO1*) were analyzed in the corollas of wild-type (black bars) and *PhCM2* RNAi lines (15, striped bars; 17, grey bars; and 23, white bars) petunia flowers harvested at 20:00 h 2 day post-anthesis. Expression values in *PhCM2* lines are presented as a percentage of corresponding transcript level in wild-type set as 100%. Data are means ± SE (n = 4 biological replicates, except n = 3 biological replicates for *PhCM2* RNAi line 23). (**b**) ADT activities were measured in plastids isolated from corollas of 1 to 3 day-old wild type (black bars) and *PhCM2* RNAi (white bars) petunia flowers (lines 15, 17, and 23) harvested at 10:00 h. Data are means ± SE. (n=3 biological replicates).

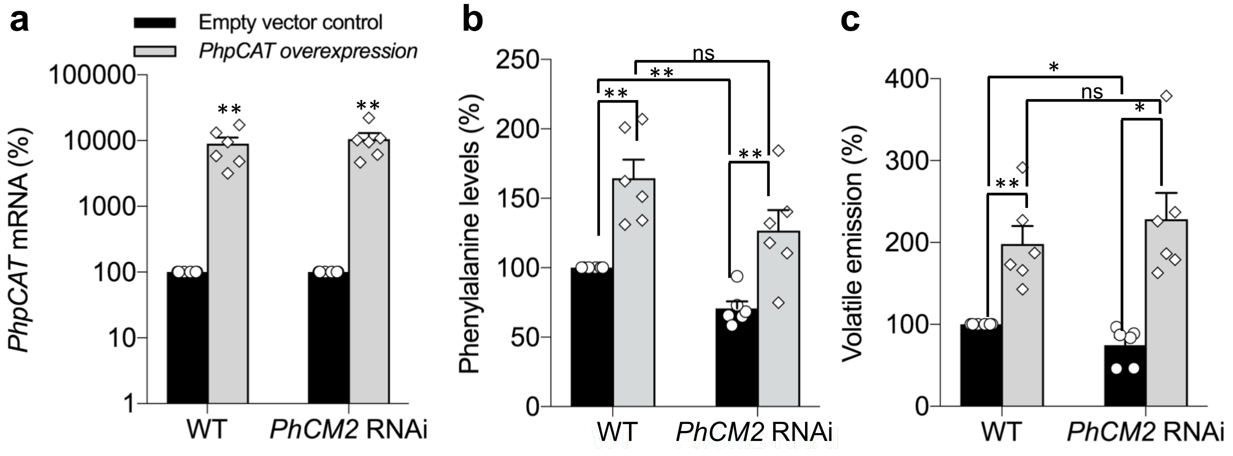

Supplementary Figure 9 | Effect of *PhpCAT* overexpression on levels of phenylalanine and phenylalanine-derived volatiles in wild-type and *PhCM2* RNAi petunia flowers.

(**a**) *PhpCAT* mRNA levels in wild type and *PhCM2* RNAi line15 petunia flowers. For each genetic background, black and grey bars represent flowers infiltrated with empty vector or the *PhpCAT* overexpression construct, respectively. Data are presented as a percentage relative to the corresponding empty-vector reference. (**b**, **c**) Levels of phenylalanine (**b**) and phenylalanine-derived volatiles (**c**) in petunia flowers of wild type and *PhCM2* RNAi line 15 infiltrated with agrobacterium carrying the empty vector (black bars) or the *PhpCAT* overexpression construct (grey bars). For **b** and **c**, data are presented as a percentage relative to the WT empty vector reference. Data are means ± SE (n = 6 biological replicates). ns, not significant; * $P < 0.05$ and ** $P < 0.01$ as determined by paired two-tailed Student's *t*-test.

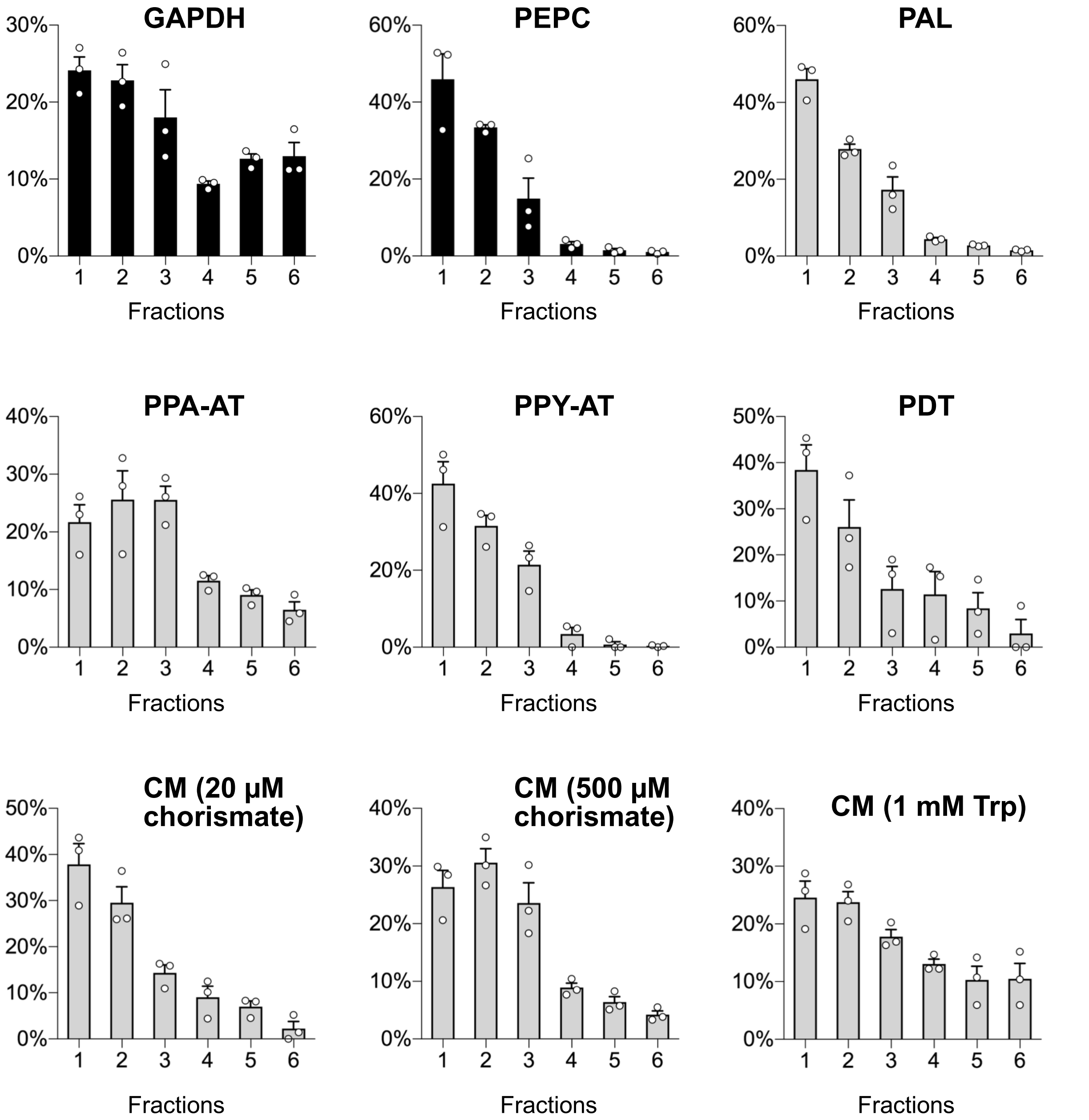

Supplementary Figure 10 | Distribution of enzyme activities across the six fractions of decreasing density obtained by non-aqueous fractionation of petunia petals. Data are presented as the percentage of total activity recovered across all fractions and are the mean ± S.E. of activities from three independent fractionations (n = 3). GAPDH, NADPH-dependent glyceraldehyde-3-phosphate dehydrogenase; PEPC, phospho(*enol*)pyruvate carboxylase; PAL, phenylalanine ammonia lyase; PPA-AT, prephenate aminotransferase; PDT, prephenate dehydratase; PPY-AT, phenylpyruvate aminotransferase; CM (20 µM), chorismate mutase assayed with 20 µM substrate; CM (500 µM), chorismate mutase assayed with 500 µM substrate; CM (1 mM Trp), chorismate mutase assayed with 500 µM substrate in the presence of 1 mM tryptophan.

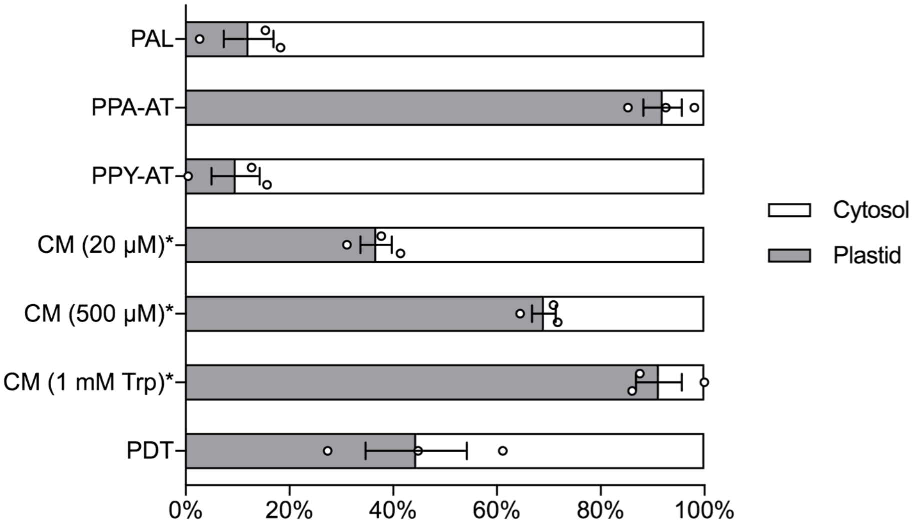

Supplementary Figure 11 | Relative distribution of enzyme activities between plastids and cytosol, as determined by non-aqueous fractionation.

Plastidic and cytosolic fractions are shown in grey and white, respectively. Data are means ± SE (n=3). PAL, phenylalanine ammonia lyase; PPA-AT, prephenate aminotransferase; PPY-AT, phenylpyruvate aminotransferase; CM, chorismate mutase; PDT, prephenate dehydratase.

*Distribution of chorismate mutase activities was determined at substrate concentrations of 20 µM and 500 µM, and at 500 µM substrate with 1 mM tryptophan, as indicated. PDT activity was measured at 2 mM of prephenate.

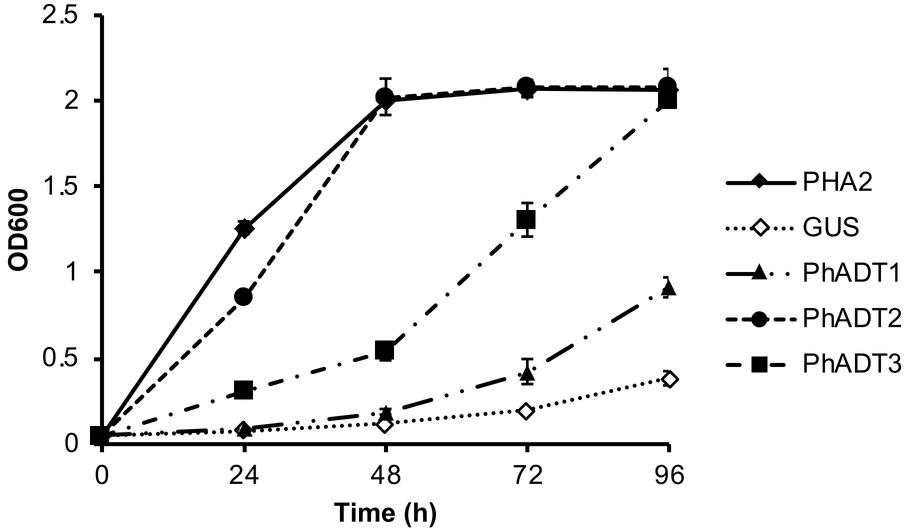

Supplementary Figure 12 | Expression of *PhADTs* in a *pha2 S. cerevisiae* mutant. The *pha2 S. cerevisiae* mutant was transformed with expression vectors carrying *PhADT1* (solid triangles), *PhADT2* (solid circles), *PhADT3* (solid squares), *S. cerevisiae PHA2* ORF (solid diamonds, a positive control) and GUS (open diamonds, a negative control) under the control of the GAL promoter. The growth of the *pha2* strain is phenylalanine dependent. All strains were first grown to stationary phase in raffinose media containing phenylalanine, then transferred at an initial OD600 of 0.05 to galactose media lacking phenylalanine and grown with shaking at 30°C for 4 days. All data are means ± SE of three independent cultures.

a

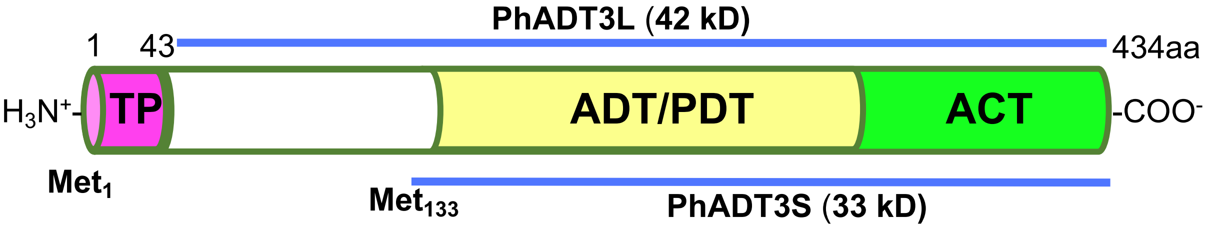

b

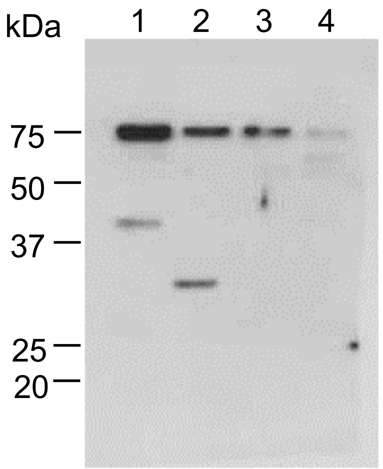

Supplementary Figure 13 | Immunodetection of the cytosolic PhADT3 isoform. (**a**) Structure of the PhADT3 protein showing positions of plastidial transit peptide (TP, pink), arogenate/prephenate dehydratase (ADT/PDT, yellow) catalytic domain, and ACT regulatory amino acid binding domain (green) according to [1]. Molecular mass of mature PhADT3L and PhADT3S isoforms are shown. (**b**) Specificity of anti-PhADT3 antibodies. Representative immunoblot using anti-PhADT3 antibodies against purified recombinant mature PhADT3L (lane 1) and PhADT3S (lane 2) isoforms, preparations from *E. coli* carrying empty pET28 expression vector (lane 3) and mature PhADT1 protein (lane 4). 1 µg of total protein was loaded in each lane.

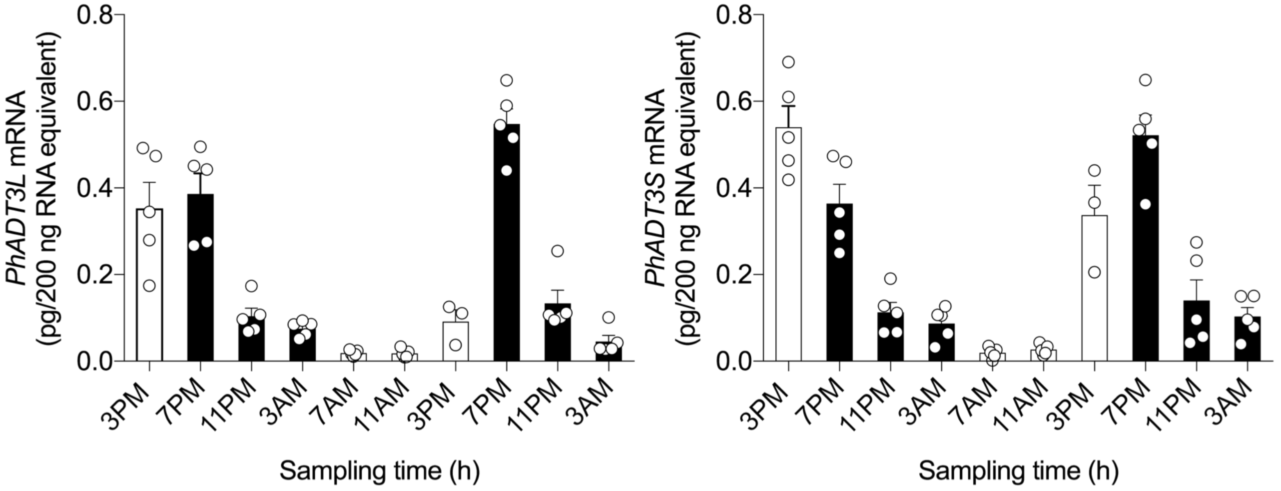

Supplementary Figure 14 | Expression profiles of *PhADT3L* and *PhADT3S* transcripts in petunia flowers over a daily light/dark cycle.
Changes in *PhADT3* transcript levels during a normal light/day cycle in petunia corolla harvested at 15:00 h on day 1 to 03:00 h on day 3 post-anthesis. *PhADT3L* mRNA levels were determined using primer set 5 (Fig. 5b) while *PhADT3S* mRNA levels were determined by subtracting the quantity of mRNA measured with primer set 5 from that of primer set 6 (Fig. 5b), as primer set 5 detects only *PhADT3L* and primer set 6 detects the entire *PhADT3* transcript population (*PhADT3L* plus *PhADT3S*). Black and white bars correspond to night and day sampling points, respectively. All data are means ± SE (n = 5 biological replicates, except for 15:00 h on day 2 post-anthesis n = 3).

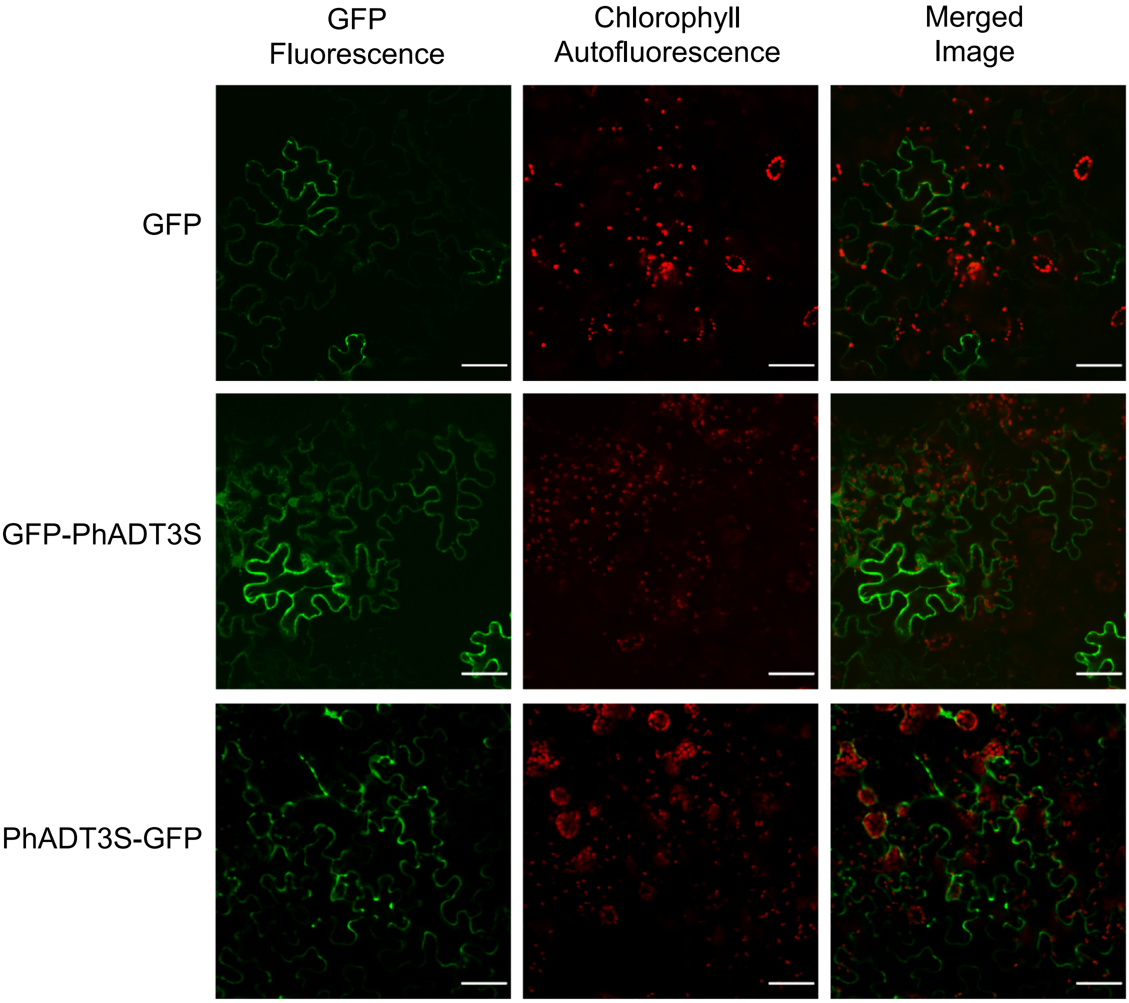

Supplementary Figure 15 | Subcellular localization of PhADT3S. *PhADT3S* was transiently expressed in *Nicotiana benthamiana* leaves with either an N-terminal (GFP-PhADT3S) or C-terminal (PhADT3S-GFP) fused GFP tag. GFP fluorescence and chlorophyll autofluorescence are shown (*Left* and *Center* panels, respectively), while the merged panels (*Right*) show the overlay of GFP and chlorophyll autofluorescence. GFP alone and chlorophyll autofluorescence were used as cytosolic and plastidic markers, respectively. Scale bars, 50 μm.

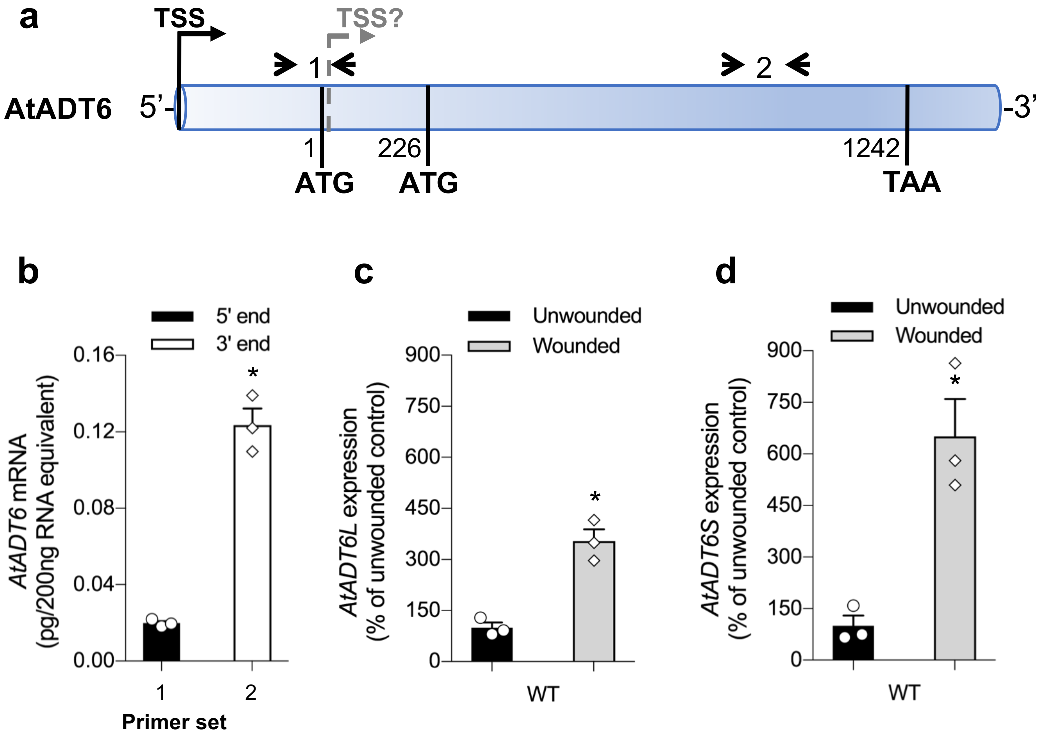

Supplementary Figure 16 | Potential alternative *AtADT6* transcripts.
(**a**) Schematic presentation of *AtADT6* mRNA and positions of gene-specific primer sets shown in Supplementary Table 1. The translation initiation codons as well as stop codons are indicated in bold. Nucleotide positions are referred to the first translation initiation codon. TTS, transcription start sites; TSS?, predicted TSS2. (**b**) The absolute levels of *AtADT6* transcripts were determined at 5′ end (black) and 3′ end (white) of mRNAs by qRT-PCR and are shown as pg/200 ng total RNA. Data are means ± SE (n=3 biological replicates). (**c**, **d**) *AtADT6L* (**c**) and *AtADT6LS* (**d**) transcript levels in wild type Arabidopsis leaves upon mechanical wounding (grey bars), as determined by qRT-PCR. Data are presented as a percentage of *AtADT6L* and *AtADT6S* transcripts in unwounded leaves (black bars), each respectively set as 100%. Data are means ± SE (n = 3 biological replicates). * $P < 0.05$ as determined by paired two-tailed Student's *t*-test.

Supplementary Table 1 | Primers used in this work

Primer Name	Sequence	Purpose
AtCM2 LB GSP	5'-GGATTCGGGTTCTGGTTGTT-3'	Genotyping
AtCM2 RB GSP	5'-TCGCAGGCTCGTAATCTTGT-3'	Genotyping
p745	5'-AACGTCCGCAATGTGTTATTAAGTTGTC-3'	Genotyping
o8409	5'-ATATTGACCATCATACTCATTGC-3'	Genotyping
AtCM2 F	5'-GGGAGCTCCATATGGCAAGAGTCTTCGAATCGGA-3'	RT-PCR
AtCM2 R	5'-CCCTCGAGGCATGCTCAATCGAGACGACGTAGAA-3'	RT-PCR
AtUbc F	5'-ATGCAGGCATCAAGAGCG-3'	RT-PCR
AtUbc R	5'-TCATCCTTTCTTAGGCATAGCG-3'	RT-PCR
AtCM2 qRT F	5'-GAAAGCAGAAACGTTTGGACAA-3'	qRT-PCR
AtCM2 qRT R	5'-AGATGCGAGAGGCAAGCAAT-3'	qRT-PCR
AtUbc qRT F	5'-CTGCGACTCAGGGAATCTTCTAA-3'	qRT-PCR
AtUbc qRT R	5'-TTGTGCCATTGAATTGAACCC-3'	qRT-PCR
AtADT6 qRT F1	5'-ACATGTTTTTCTACATTCTCGACTTTG-3'	qRT-PCR
AtADT6 qRT R1	5'-CCGGCTGGCTAGCACCTAA-3'	qRT-PCR
AtADT6 qRT F2	5'-AGGAACCAGCGTGCTTTTCA-3'	qRT-PCR
AtADT6 qRT R2	5'-CCTGATCGGACGGTTGTGAT-3'	qRT-PCR
PhCM2 RNAi F	5'-CTCGAGTCTAGAATCAAATTCCCAATAAATTCCACC-3'	Cloning
PhCM2 RNAi R	5'-GAATTC GGATCCTGAATATCACAGGCAGCAGT-3'	Cloning
PhCM1 F	5'-CATATGCAAGCTTCTGCAACTTCTC-3'	Cloning
PhCM1 R	5'-GGATCCCTAATCCAGTCTTCTCAG-3'	Cloning
PhCM2 F	5'-CATATGGCCTGTGGTGATTATG-3'	Cloning
PhCM2 R	5'-GGATCCCAAACTAACTTCTATCTAA-3'	Cloning
PhADT3S F	5'-CATATGATGCACGGGGCGCAGCT-3'	Cloning
PhADT3S R	5'-GGATCCTTAAGCATCCCTGGAAGG-3'	Cloning
PhADT1 GTW F	5'-AAAAAGCAGGCTTCATGCAGTCCCTTACTCCT-3'	Gateway Cloning
PhADT1 GTW NS R	5'-AGAAAGCTGGGTCTTCATCCCTAGAAGGACAG-3'	Gateway Cloning
PhADT2 GTW F	5'-AAAAAGCAGGCTTCATGGCAGCCACCACTACAC-3'	Gateway Cloning
PhADT2 GTW NS R	5'-AGAAAGCTGGGTCAGCTATTCCACTATCTGAC-3'	Gateway Cloning
PhADT3 GTW F	5'-AAAAAGCAGGCTTCATGCAGTCCCTTACTCCATC-3'	Gateway Cloning
PhADT3S GTW F	5'-AAAAAGCAGGCTTCATGCACGGGGCGCAGCTG-3'	Gateway Cloning
PhADT3 GTW NS R	5'-AGAAAGCTGGGTCAGCATCCCTGGAAGGAGAC-3'	Gateway Cloning
PhADT3 GTW R	5'-AGAAAGCTGGGTCTTAAGCATCCCTGGAAGGA-5'	Gateway Cloning
PhUBQ qRT F	5'-GTTAGATTGTCTGCTGTCGATGGT-3'	qRT-PCR
PhUBQ qRT R	5'-AGGAGCCAATTAAAGCACTTATCAA-3'	qRT-PCR
PhEF1-α F	5'-CCTGGTCAAATTGGAAACGG-3'	qRT-PCR
PhEF1-α R	5'-CAGATCGCCTGTCAATCTTGG-3'	qRT-PCR
PhCM1 qRT F	5'-CCTGCTGTTGAAGAGGCTATCA-3'	qRT-PCR

PhCM1 qRT R	5'-CAGGGTCACCTCCATTTTCTG-3'	qRT-PCR
PhCM2 qRT F	5'-TGCAACTACTGCTGCCTGTGAT-3'	qRT-PCR
PhCM2 qRT R	5'-TCGTCAGAGCAATCCCTGAAT-3'	qRT-PCR
DAHPS qRT F	5'-CAAAGCTCCGTGTGGTCTTAAA-3'	qRT-PCR
DAHPS qRT R	5'-TCCTGGGTGGCTTCCTTCTT-3'	qRT-PCR
EPSPS qRT F	5'-CACCCCACCGGAGAAACTAA-3'	qRT-PCR
EPSPS qRT R	5'-TGACGGGAACATCTGCACAA-3'	qRT-PCR
ODO1 qRT F	5'-ATTCGCCATGGGAATTTCC-3'	qRT-PCR
ODO1 qRT R	5'-GAAAGTGTCTTCCCAGGATGTCA-3'	qRT-PCR
PhADT1 qRT F1	5'-GAGCTCCGGCGAGTTACAAC-3'	qRT-PCR
PhADT1 qRT R1	5'-GTGTCCGGCTGCTGTGAGA-3'	qRT-PCR
PhADT1 qRT F2	5'-TAACTGCGAAGCCATTCCCTGC-3'	qRT-PCR
PhADT1 qRT R2	5'-CTCTACTGGTAGAACTGCGCG-3'	qRT-PCR
PhADT2 qRT F3	5'-ACCATCTCATCTCCCGCTCTT-3'	qRT-PCR
PhADT2 qRT R3	5'-TGTAGTGGTGGCTGCCATTG-3'	qRT-PCR
PhADT2 qRT F4	5'-ACGAAGTTGGGTTTGGTCAG-3'	qRT-PCR
PhADT2 qRT R4	5'-TGCCCCTGCATCTTTTAGTT-3'	qRT-PCR
PhADT3 qRT F5	5'-AGCAGCCGCCGTAAACAC-3'	qRT-PCR
PhADT3 qRT R5	5'-TGCACACGAACTCTGCCAAT-3'	qRT-PCR
PhADT3 qRT F6	5'-CAAAATGTGAAGCTATTCCTTGTG-3'	qRT-PCR
PhADT3 qRT R6	5'-TTCGATCGGTAAAACAGCACG-3'	qRT-PCR
PhADT2 GSP	5'-TGCCCCTGCATCTTTTAGTT-3'	5' RACE
PhADT3 GSP	5'-GCTCTTACGTCCGCCATTGACGCT-3'	5' RACE

Supplementary Reference

1. Zhang, S. et al. Chorismate mutase-prephenate dehydratase from Escherichia coli study of catalytic and regulatory domains using genetically engineered proteins. *J. Biol. Chem.* **273**, 6248-6253 (1998).
