# Supplementary material for: Completion of the cytosolic post-chorismate phenylalanine biosynthetic pathway in plants
Source: Nat Commun. 2019 Jan 3;10:15. doi: 10.1038/s41467-018-07969-2 (PMC6318282; doi:10.1038/s41467-018-07969-2)
Supplement: Supplementary file 3 — Reporting Summary [file 41467_2018_7969_MOESM3_ESM.pdf]

Corresponding author(s): Natalia Dudareva

Reporting Summary

Nature Research wishes to improve the reproducibility of the work that we publish. This form provides structure for consistency and transparency in reporting. For further information on Nature Research policies, see Authors & Referees and the Editorial Policy Checklist.

Statistical parameters

When statistical analyses are reported, confirm that the following items are present in the relevant location (e.g. figure legend, table legend, main text, or Methods section).

n/a	Confirmed	
☐	☒	The exact sample size (*n*) for each experimental group/condition, given as a discrete number and unit of measurement
☐	☒	An indication of whether measurements were taken from distinct samples or whether the same sample was measured repeatedly
☐	☒	The statistical test(s) used AND whether they are one- or two-sided<br>*Only common tests should be described solely by name; describe more complex techniques in the Methods section.*
☐	☒	A description of all covariates tested
☒	☐	A description of any assumptions or corrections, such as tests of normality and adjustment for multiple comparisons
☐	☒	A full description of the statistics including central tendency (e.g. means) or other basic estimates (e.g. regression coefficient) AND variation (e.g. standard deviation) or associated estimates of uncertainty (e.g. confidence intervals)
☐	☒	For null hypothesis testing, the test statistic (e.g. *F*, *t*, *r*) with confidence intervals, effect sizes, degrees of freedom and *P* value noted<br>*Give P values as exact values whenever suitable.*
☒	☐	For Bayesian analysis, information on the choice of priors and Markov chain Monte Carlo settings
☒	☐	For hierarchical and complex designs, identification of the appropriate level for tests and full reporting of outcomes
☒	☐	Estimates of effect sizes (e.g. Cohen's *d*, Pearson's *r*), indicating how they were calculated
☐	☒	Clearly defined error bars<br>*State explicitly what error bars represent (e.g. SD, SE, CI)*

Our web collection on statistics for biologists may be useful.

Software and code

Policy information about availability of computer code

Data collection	GC-MS data were collected using MSD Chemstation v.E.01.00.237. LC-MS data were collected using ChemStation LC and CE Drivers version A.01.13 [014]. qRT-PCR data were collected using StepOne v.2.2.2.
Data analysis	GC-MS data were analyzed using GraphPad Prism v6.05. Matlab R2013a was used for metabolic flux analysis.

For manuscripts utilizing custom algorithms or software that are central to the research but not yet described in published literature, software must be made available to editors/reviewers upon request. We strongly encourage code deposition in a community repository (e.g. GitHub). See the Nature Research guidelines for submitting code & software for further information.

Data

Policy information about availability of data

All manuscripts must include a data availability statement. This statement should provide the following information, where applicable:

- Accession codes, unique identifiers, or web links for publicly available datasets
- A list of figures that have associated raw data
- A description of any restrictions on data availability

Raw data for all Figures containing experimental data are provided in a Source data file. Proteomic raw data and MaxQuant search results have been made publicly available through MassIVE (https://massive.ucsd.edu/) with submission ID: MSV000083169.

Field-specific reporting

Please select the best fit for your research. If you are not sure, read the appropriate sections before making your selection.

☒ Life sciences ☐ Behavioural & social sciences ☐ Ecological, evolutionary & environmental sciences

For a reference copy of the document with all sections, see nature.com/authors/policies/ReportingSummary-flat.pdf

Life sciences study design

All studies must disclose on these points even when the disclosure is negative.

Sample size	A minimum sample size of 3 independent biological experiments was chosen to obtain valid statistical analyses.
Data exclusions	No data exclusion
Replication	At least three biological replicates were performed for each experiment. All attempts at replication were successful and all data included in the manuscript.
Randomization	Plants were randomized in greenhouse, and sample orders were randomized during analysis.
Blinding	Blinding is not necessary because results are quantitative for all experiments, and not subjective. Blinding was not possible in these experiments, as samples were analyzed immediately following treatments, and thus the treatment was known to the investigator.

Reporting for specific materials, systems and methods

Materials & experimental systems

n/a	Involved in the study	
☐	☒	Unique biological materials
☐	☒	Antibodies
☒	☐	Eukaryotic cell lines
☒	☐	Palaeontology
☒	☐	Animals and other organisms
☒	☐	Human research participants

Methods

n/a	Involved in the study	
☒	☐	ChIP-seq
☒	☐	Flow cytometry
☒	☐	MRI-based neuroimaging

Unique biological materials

Policy information about availability of materials

Obtaining unique materials	Seeds of transgenic petunia plants generated for this study are available upon request.

Antibodies

Antibodies used	Rabbit anti-PhADT3 polyclonal antibodies were generated against a synthetic PhADT3 peptide CFKTSIVFAHDKGTS and purified by cross-absorption with PhADT1 specific peptide CFKTSIVFAHEGTGV to increase antibodies specificity and prevent cross-reactivity with PhADT1 (Genscript, Piscataway, NJ). Pierce™ goat anti-rabbit IgG (H+L) horseradish peroxidase conjugate was used as secondary antibodies (Catalog # 31460, Thermo Scientific).
Validation	Rabbit anti-PhADT3 polyclonal antibodies were validated in the manuscript (Supplementary Figure 13).
